# PARP1 depletion improves mitochondrial and heart function in Chagas disease: Effects on POLG dependent mtDNA maintenance

**DOI:** 10.1371/journal.ppat.1007065

**Published:** 2018-05-31

**Authors:** Jake Jianjun Wen, Yuhui Whitney Yin, Nisha Jain Garg

**Affiliations:** 1 Department of Microbiology and Immunology, University of Texas Medical Branch, Galveston, TX, United States of America; 2 Department of Pharmacology & Toxicology, University of Texas Medical Branch, Galveston, TX, United States of America; 3 Department of Pathology, University of Texas Medical Branch, Galveston, TX, United States of America; 4 Institute for Human Infections and Immunity, University of Texas Medical Branch, Galveston, TX, United States of America; U Tex SouthWestern, UNITED STATES

## Abstract

Chagasic cardiomyopathy is caused by *Trypanosoma cruzi* infection. Poly(ADP-ribose) polymerase 1 (PARP1) is known for its function in nuclear DNA repair. In this study, we have employed genetic deletion and chemical inhibition approaches to determine the role of PARP1 in maintaining mtDNA dependent mitochondrial function in Chagas disease. Our data show that expression of PARP1 and protein PARylation were increased by >2-fold and >16-fold, respectively, in the cytosolic, nuclear, and mitochondrial fractions of the human cardiac myocytes and the myocardium of wildtype (WT) mice chronically infected with *T*. *cruzi*. The nuclear and cytosolic PARP1/PAR did not interfere with the transcription and translation of the components of the mtDNA replisome machinery in infected cardiomyocytes and chagasic murine myocardium. However, PARP1 binding to Polymerase γ and mtDNA in mitochondria were increased, and associated with a loss in mtDNA content, mtDNA-encoded gene expression, and oxidative phosphorylation (OXPHOS) capacity, and an increase in mitochondrial ROS production in cells and heart of WT mice infected with *T*. *cruzi*. Subsequently, an increase in oxidative stress, and cardiac collagen deposition, and a decline in LV function was noted in chagasic mice. Genetic deletion of PARP1 or treatment with selective inhibitor of PARP1 (PJ34) improved the mtDNA content, mitochondrial function, and oxidant/antioxidant balance in human cardiomyocytes and chronically infected mice. Further, PARP1 inhibition was beneficial in preserving the cardiac structure and left ventricular function in chagasic mice. We conclude that PARP1 overexpression is associated with a decline in Pol γ-dependent maintenance of mtDNA content, mtDNA-encoded gene expression, and mitochondrial respiratory function, and subsequently contributes to an increase in mtROS and oxidative stress in chagasic myocardium. Inhibition of mitochondrial PARP1/PAR offers a novel therapy in preserving the mitochondrial and LV function in chronic Chagas disease.

## Author summary

Chagasic cardiomyopathy is caused by *Trypanosoma cruzi* infection, and it affects ~7 million individuals on the American continent. Poly(ADP-ribose) polymerase 1 (PARP1) is a nuclear DNA repair enzyme. In this study, we demonstrate that PARP1 tends to be localized to mitochondria in chagasic myocardium. The mtPARP1 was bound to mitochondrial DNA polymerase gamma (Pol γ) that is essential for mtDNA synthesis and repair. Genetic or chemical inhibition of PARP1 was beneficial in improving the mtDNA content, and mitochondrial and left ventricular (LV) function in Chagas disease. Our results suggest that mitochondrial transport of PARP1 adversely impacts the mtDNA maintenance by Pol γ replisome, and exacerbates the mitochondrial dysfunction, oxidative stress, and cardiac remodeling in Chagas disease. We propose that small molecules that prevent PARP1 transport to mitochondria or that arrest PARP1 effects on Pol γ activity will be beneficial in preserving the mitochondrial health and LV function in chronic cardiomyopathy of chagasic (and potentially other) etiologies.

## Introduction

Chagas disease, caused by *Trypanosoma cruzi* (*T*. *cruzi* or *Tc*) infection, affects ~7 million individuals on the American continent [1]. In the US, autochthonous and congenital transmission of *Tc* occurs, and >300,000 individuals are infected with *Tc*, resulting in increased risk of transmission through blood and organ donation [2]. The presentation of chagasic cardiomyopathy and heart failure in infected individuals results in >17,000 deaths per year and costs >$8.0 billion per year in health care costs and lost productivity [3]. The currently available anti-parasite drugs cause significant toxicity and therapeutic failure in adults, and are not recommended [4]. New druggable targets that can halt the destructive oxidative and inflammatory processes and thereby arrest the cardiac remodeling and heart failure in Chagas disease are urgently needed.

Studies in experimental models and humans have demonstrated that the infected host sustains oxidative stress in the myocardium (reviewed in [5,6]). *In vivo* studies in experimental models showed that mitochondrial functional defects of respiratory chain complexes contribute to a compromise in oxidative phosphorylation (OXPHOS) capacity and an increase in mitochondrial reactive oxygen species (mtROS) in chronic Chagas disease [7–9]. Recent studies also noted the mitochondrial dysfunction and pro-oxidant milieu were presented with peripheral and myocardial increase in protein carbonyls and lipid hydroperoxides (LPO) in chagasic humans [10–12]. Enhancing the mtROS scavenging capacity by overexpressing Mn^+2^ superoxide dismutase (MnSOD) improved the left ventricular (LV) function that otherwise was significantly compromised in chronically infected WT mice [13]. These studies demonstrate pathologic significance of mtROS in chagasic cardiomyopathy. Why mitochondrial defects persist in chagasic heart is not known.

The integrity and replication of mtDNA is essential for mitochondrial health. The mtDNA is synthesized by DNA polymerase γ (Pol γ) that consists a 140-kDa catalytic subunit (encoded by *POLG*) and two 55-kDa processive subunits (encoded by POLG2) [14]. The Pol γ possesses DNA polymerase activity as well as 3’-5’ exonuclease activity for proofreading, and functions in conjunction with a number of additional mitochondrial proteins, e.g., 5’-3’ DNA helicase (also called Twinkle, TWNK), topoisomerase I (TOP1MT), RNA polymerase (POLRMT), RNase HI (RNASEH1), exonuclease 1 (MGME1), single-stranded DNA binding protein 1 (SSBP1), DNA ligase III (LIG3), DNA helicase/nuclease 2 (DNA2), and RNA and DNA flap endonuclease 1 (FEN1), forming the mtDNA replisome. The TWNK unwinds the dsDNA; mtSSB stabilizes the single strands; and TOP1MT releases the torsional tension of mtDNA ahead of the replication fork. In addition, POLRMT and transcription factor A (TFAM) are required for mitochondrial transcription, and also provide RNA primer that initiates Pol γ dependent DNA replication. DNA primase-polymerase (PrimPol) contains both DNA primase and DNA polymerase activities, is present in both nuclear and mitochondrial compartments, and it can play an important role in re-priming DNA synthesis to rescue a stalled replication fork [15].

ROS-induced single strand breaks in DNA and oxidation of guanine to 8-oxoguanine (8-oxoG) signal the activation of members of the poly(ADP-ribose) polymerase (PARP) family to facilitate DNA repair [16]. The 113-kDa PARP1 protein (89-kDa active form) belongs to the PARP family of 7 known and 10 putative members, and it accounts for >85% of the PARP activity in cellular systems. PARP1 catalyzes the cleavage of NAD^+^ into nicotinamide and ADP-ribose and uses the latter to synthesize PAR polymers. The basal level activation of PARP1 by mild genotoxic stimuli causes PARylation of histone proteins (e.g. H_1_ and H_2_B) that mediates relaxation of the chromatin superstructure and recruitment of DNA-break repair enzymes, resulting in DNA repair and cell survival. Significant research efforts have shown that PARP1 functions in chromosomal DNA repair through PARylation of histone proteins [17]. It is also suggested that PARP1 by direct binding to or PARylation of enhancers and promoters, functions as a transcriptional co-activator, and modulates the expression of self and many other genes [18]. The scientific community has; however, largely overlooked the potential role of PARP1 in maintenance of mtDNA in health and disease.

Recently, we have found that mitochondrial transport of PARP1 and PARylation were increased in *Tc*-infected cardiomyocytes [19]. In this study, we aimed to determine the role of PARP1 in mitochondrial health during Chagas disease. For this, we employed wild type (WT or PARP1^+/+^), PARP1^+/-^, and PARP1^-/-^ mice. In some studies, we also treated WT chagasic mice with a selective PARP1 inhibitor PJ34 [20]. We utilized fluorescence probes, biochemical and molecular assays, and cutting-edge *in situ* respirometry and 3-dimensional echocardiography to test our hypothesis. Our results suggest that genetic deletion or chemical inhibition of PARP1 was beneficial in improving mitochondrial health in chagasic mice. Genetic depletion or chemical inhibition of PARP1 resulted in a decline in chronic oxidative stress and cardiac remodeling, and an improvement in mitochondrial coupled respiration and LV function. We discuss the potential mechanism of PARP1/PAR interference with mitochondrial function in Chagas disease.

## Materials and methods

### Ethics statement

All animal experiments were performed according to the National Institutes of Health Guide for the Care and Use of Laboratory Animals, and were approved by the Institutional Animal Care and Use Committee at the University of Texas Medical Branch, Galveston (protocol number: 805029).

### Mice, parasites, and cell culture

129S-PARP1^tm1Zqw^/J (PARP1^-/-^) mice were crossed with C57BL/6 mice to generate PARP1^-/-^ mice on 129S/BL6 genetic background. PARP1^-/-^ mice were bred with WT mice to generate PARP1^+/-^ mice. All breeding pairs were purchased from Jackson Laboratory (Bar Harbor ME), and a standard PCR was performed to confirm the genotype of WT (PARP1^+/+^), PARP1^+/-^ and PARP1^-/-^ mice (S1A Fig).

*T*. *cruzi* (SylvioX10/4, ATCC 50823) was propagated by *in vitro* passage in C2C12 cells. The WT, PARP1^+/-^ and PARP1^-/-^ mice (all 129S/BL6 background, 6-weeks-old) were infected with *T*. *cruzi* (10,000 trypomastigotes/mouse, intraperitoneal). For some studies, C57BL/6 mice were infected as above, and then mice were given a treatment of 2-(dimethylamino)-N-(6-oxo-5,6-dihydrophenanthridin-2-yl)acetamide hydrochloride (PJ34, Sigma-Aldrich, St Louis MO). PJ34 is a cell-permeable, water-soluble, selective PARP1 inhibitor PJ34 (EC_50_ = 20 nM) and shown to be ~10,000 times more potent than the prototypical PARP inhibitor, 3-aminobenzamide [20,21]. PJ34 (12.5 mg/kg) was delivered intraperitoneally for three weeks (twice a week) beginning at 45 days’ post-infection (pi) when acute parasitemia was controlled. All mice were harvested at 150 days’ pi corresponding to chronic disease phase. Sera/plasma and tissue samples were stored at 4°C and -80°C, respectively.

Human cardiomyocyte cells (AC16, cat#SCC109, EMD Millipore, Burlington MA) were cultured and maintained in Dulbecco’s modified Eagle’s medium (DMEM)/F-12 medium containing 12.5% fetal bovine serum (FBS). Human cervix epithelial cells (HeLa, ATCC, Manassas VA) were propagated in DMEM media supplemented with Earle's salts, 2 mM L-glutamine and 10% FBS. Cells were infected with *T*. *cruzi* (cell: parasite ratio: 1:5) for various studies.

### Gene expression analysis

Freshly harvested heart tissue sections (10 mg) were snap-frozen in liquid nitrogen and homogenized in TRIzol reagent (Invitrogen, Carlsbad, CA; weight/volume ratio, 1:10). Freshly cultured cells (10^6^/well, 6-well plates) were directly homogenized in TRIzol reagent. Total RNA was extracted by chloroform/isopropanol/ethanol method, treated with RNase free DNase I (NEB, Beverly MA), and assessed for quality (OD_**260/280**_ ratio ≥ 2.0) and quantity (OD_260_ of 1 = 40 μg/ml RNA). Total RNA (2 μg) was reverse transcribed by using poly (dT)18 oligonucleotide with an iScript kit (Bio-Rad, Hercules CA). The cDNA was utilized as a template, and quantitative real-time PCR was performed on an iCycler Thermal Cycler with SYBR-Green supermix (Bio-Rad) and gene-specific oligonucleotide pairs. The PCR Base Line Subtracted Curve Fit mode was applied for threshold cycle (Ct), Ct values for target mRNAs were normalized to GAPDH mRNA, and the relative expression level of each target gene was calculated as 2^−ΔCt^ (ΔC_t_ = C_t sample_—C_t control_) [22]. The oligonucleotide pairs used for amplifying the mRNAs are listed in Table A in S1 Table.

### Tissue and cell lysis and fractionation

Freshly harvested tissues (tissue: buffer ratio, 1:10 w/v) were homogenized in RIPA buffer (cat# 9806, Cell Signaling, Dallas TX), centrifuged at 10,000 g, and supernatants used as heart tissue lysates. Sub-organelle fractions were simultaneously prepared by following the published protocol [23]. Briefly, tissues were suspended in homogenization buffer (50 mM Tris-HCl, pH 7.4, 5 mM MgCl_2_, 1 mM DTT, 25 μg/ml spermine, 25 μg/ml spermidine, and protease inhibitor cocktail) containing 250 mM sucrose; tissue: buffer ratio, 1:20) and homogenized at 4°C by using a dounce homogenizer. The homogenates were centrifuged at 800 g for 15 min at 4^o^ C, and supernatants and pellet collected. The pellets were re-solubilized in four volumes of homogenization buffer containing 2 M sucrose by repeated pipetting, homogenized with a single stroke of the dounce homogenizer, filtered to remove debris, layered on top of 4 ml cushion of homogenization buffer/2M sucrose, and centrifuged for 35 min in a swing-bucket ultracentrifuge at 80,000 g. After aspirating the supernatant, pellets containing pure nuclei were stored at -70°C. The supernatants from low speed centrifugation step were centrifuged at 6000 g for 15 min, and resultant pellets and supernatants were stored as mitochondrial and cytosolic fractions, respectively. Markers of nuclear (Lamin B), and mitochondrial (COIV subunit) fractions were evaluated in all mitochondrial fractions, and mitochondrial fractions that exhibited > 6% of contaminant were re-centrifuged as described above to ensure purity. The mitochondrial fractions were also confirmed for purity by PCR evaluation of nuDNA encoded *GAPDH* fragment and mtDNA encoded *7S* fragment (S1B Fig).

Cardiomyocyte or HeLa cells were seeded in 6-well plates (1x10^6^ cells per well), and incubated with *T*. *cruzi* (cell/parasite ratio of 1:5) in presence or absence of 1 μM PJ34 for 24 h. Cells were suspended in lysis buffer (50 mM Tris pH 7.5, 150 mM NaCl, 1 mM EDTA, 1 mM EGTA, 1% Nonidet P-40, 2.5 mM KH_2_PO_4_, and 1 mM Na_3_VO_4_), and incubated on ice for 30 min. Cell lysates were centrifuged at 12,000 g at 4°C for 15 min and the resultant supernatants were stored at −80°C. For fractionation, cells (7×10^6^/ml) were incubated on ice for 30 minutes in buffer A (10 mM HEPES, pH 7.9, 10 mM NaCl, 0.1 mM EDTA, 0.1 mM EGTA, 1 mM DTT, 1 mM PMSF) containing 0.625% NP-40 and 1% protease inhibitor cocktail. Cell lysates were centrifuged at 4°C at 10,000 g for 1 min and supernatants were stored as a cytosolic fraction. Pellets were washed with buffer A containing 1.7 M sucrose, re-suspended in buffer B (20 mM HEPES pH 7.9, 0.4 M NaCl, 1 mM EDTA, 1 mM EGTA, 1 mM DTT, and 1 mM PMSF), and centrifuged at 4°C at 13,000 g for 5 minutes. The resultant supernatants were stored at −80°C as nuclear extracts. Mitochondria were isolated from cells as above.

### Western blotting

Heart and cell homogenates (30 μg protein), or isolated cytosolic (20 μg protein), mitochondrial (15 μg protein), and nuclear (20 μg protein) fractions were electrophoresed on a 4–15% Mini-Protein TGX gel using a Mini-PROTEAN electrophoresis chamber (Bio-Rad), and proteins were transferred to a PVDF membrane using a Criterion Trans-blot System (Bio-Rad). Membranes were blocked with 5% non-fat dry milk (NFDM) in 50 mM Tris-HCl (pH 7.5) / 150 mM NaCl (TBS), washed with TBS-0.1% Tween 20 (TBST) and TBS, and incubated overnight at 4°C with antibody clones listed in Table C of S1 Table. Antibodies from Santa Cruz were used at 1:200 dilutions, and all other antibodies were used at 1:1000 dilution in TBST-5% NFDM. Membranes were washed with TBST and TBS, incubated with HRP-conjugated secondary antibody (1: 10,000 dilution, Southern Biotech, Birmingham AL), and images were acquired by using an Image Quant LAS4000 system (GE Healthcare, Pittsburgh MA). Immunoblots were subjected to Ponceau S staining to confirm equal loading and transferring of samples. Densitometry analysis of protein bands was performed using a Fluorchem HD2 Imaging System (Alpha Innotech, San Jose CA), and normalized against GAPDH (tissue homogenates and cytosolic fractions), COIV (mitochondrial fractions) or Lamin B (nuclear fractions).

### Mitochondrial DNA and *T*. *cruzi* DNA

Heart tissue sections (10 mg) were subjected to Proteinase-K lysis and total DNA was extracted by phenol/chloroform extraction/ethanol precipitation method. Total DNA (100 ng) was treated with RNase A (EN0531, Thermo Scientific, Waltham MA), purified by using DNeasy Mini Spin Columns (Qiagen, Germantown MD), and examined for quality (OD_260_/OD_280_ ratio of 1.7–2.0) and quantity ([OD_260_ –OD_320_] x 50-μg/ml) by using a DU 800 UV/visible spectrophotometer. To assess the cardiac mtDNA integrity, total DNA was used as template with mtDNA-specific primer sets and *PfuUltra* II Fusion HS DNA polymerase (Stratagene, La Jolla CA). The long range PCR to amplify 10 kb mtDNA was performed with hot start of 2 min@92°C followed by 15sec@92°C, 30sec@50°C, 8min@68°C for 28 cycles. Densitometry analysis of mtDNA bands was performed as above, and 10 kb mtDNA level was normalized to short-length, 117-bp mtDNA and 96-bp *GAPDH* nuDNA.

To assess tissue parasite burden, total DNA (100 ng) was used as a template with SYBR Green Supermix (1708882, Bio-Rad) and *Tc*18SrDNA-specific primers, and real-time qPCR was performed on an iCycler thermal cycler. Data were normalized to *GAPDH*, and relative parasite burden was calculated as 2^-ΔCt^ (ΔC_t_ = Ct_*Tc*18SrDNA_—Ct_GAPDH_). The primers are listed in Table B of S1 Table.

### Immuno-precipitation (IP) and Chromatin IP (ChIP)

Tissue or cell lysates (1 mg/ml protein) in IP incubation buffer (Active Motif, Carlsbad CA), were incubated overnight at 4°C with 20 μg of antibodies against POLG, PARP1, or PAR molecules. Samples were then loaded on to Protein G Agarose Prepacked Columns (Active Motif), and columns were washed five times with IP wash buffer (Active Motif), to remove non-binding proteins. Immune complexes were eluted from the columns in 0.2 M glycine (pH 2.5) solution. The eluents were pooled (total 100 μl), neutralized with 20 μl of 1 M Tris-HCl (pH 9.0), and used for Western blotting.

For ChIP assay, Imprint Chromatin Immunoprecipitation Kit was employed (Sigma). Briefly, Hela cells (5 X 10^7^) or tissue sections (10 mg) were incubated with 9 ml of 1% buffered formaldehyde for 30 min at room temperature on a rocking platform to cross-link DNA/proteins, neutralized by adding 1 ml of 1.25 M glycine buffer, homogenized, and then sonicated on a Misonix XL2020 sonicator (30 pulses, 30 sec on/off, 100% power) to fragment the chromatin DNA. Cross-linked samples were subjected to immune-precipitation with mouse hPARP1 N-terminus-specific monoclonal antibody (sc-74470, Santa Cruz). Mouse IgG (Sigma) was used as negative control. The formaldehyde cross-link was reversed by incubating for 15 min at 65°C. Samples were then treated with RNase H and protease K, to digest RNA and proteins, respectively, and DNA was extracted with phenol/chloroform and purified by using QIAquick PCR Purification Kit (28104, Qiagen). ChIP DNA was used as substrate for PCR amplification of PARP1-bound sequences by using the oligonucleotides listed in S1 Table.

### In-tissue mitochondrial function analysis

Freshly harvested tissues (~10 mg) were immersed in ice-cold BIOPS buffer (10 mM CaK_2_-EGTA, 7.2 mM K_2_-EGTA, 20 mM imidazole, 20 mM taurine, 50 mM K-MES, 0.5 mM dithiothreitol, 6.5 mM MgCl_2_, 5.8 mM ATP, and 15 mM creatine phosphate; pH 7.1). Myofiber bundles were transferred to 2 ml of MIR05 buffer (0.5 mM EGTA, 3 mM MgCl_2_, 60 mM K-lactobionate, 20 mM taurine, 10 mM KH_2_PO_4_, 20 mM HEPES, 110 mM sucrose, and 1 mg/ml fatty acid free bovine serum albumin, pH 7.1) containing 50-μg/ml saponin, and incubated at 4°C for 30 min to achieve chemical permeabilization of the sarcolemma membrane. Permeabilized myofiber bundles (~2 mg) were washed with MIR05 buffer, and used for measuring mitochondrial respiration by using an Oxygraph-2k (O2K) respirometer (Oroboros Instruments, Innsbruck Austria). Oxygen concentration was determined at 2 sec intervals and used to compute oxygen flux per mg of tissue by Oroboros DatLab software. Briefly, after recording the baseline respiration with myofiber bundles alone, 5 mM pyruvate, 2 mM malate and 10 mM glutamate (P+G+M) were added, and complex I driven state 4 respiration was recorded. Electron transfer was coupled to phosphorylation by the addition of 5 mM ADP, and state 3 respiration was recorded. Maximal state 3 respiration with parallel electron input from complex I and complex II was recorded with addition of 10 mM succinate, and complex II supported respiration was measured in presence of 6.25 μM rotenone (inhibits complex I) [22].

### ROS production, oxidative stress markers, and antioxidant capacity

To measure the mitochondrial ROS production, freshly isolated mitochondria (25-μg protein) were suspended in 50 μl of HMS medium (10 mM HEPES pH 7.4, 225 mM mannitol, 75 mM sucrose), and added in triplicate to 96-well, black flat-bottomed plates. The reaction was started with addition of 50 μl of 2X reaction buffer (20 mM Tris-HCl at pH 7.4, 500 mM sucrose, 2 mM EDTA) containing 66-μM amplex red and 0.2U/ml horseradish peroxidase. Mitochondria were energized with complex I or complex II substrates, and amplex red oxidation by ROS to fluorescent resorufin was measured for three minutes at Ex_563nm_/Em_587nm_ on a SpectraMax M5 microplate reader (Molecular Devices, Sunnyvale CA). Standard curve was prepared with H_2_O_2_ (50 nM–5 μM) [8].

To measure the H_2_O_2_ levels, 50 μl of tissue lysates (100 μg) or cell lysates (1 x 10^4^ cells) were added in triplicate to flat-bottom (dark-walled) 96-well plates. Then 100 μl of reaction mixture containing 0.05 M sodium phosphate, pH 7.4, 33 μM amplex Red, and 0.1 U/ml HRP was added. The plates were incubated for 30 min in dark, and ROS levels were recorded as above.

The level of 3-nitrotyrosine (3-NT) in heart tissue lysates was measured by using an ELISA kit (ab116691, Abcam, Cambridge MA). Protein carbonyls in tissue homogenates and plasma were measured by a colorimetric protein carbonyl assay (cat#10005020, Cayman Chemical, Ann Arbor MI). Malonyldialdehydes (MDA) provide a measure of lipid peroxidation products, and were measured in tissue lysates and plasma samples by a TBARS assay (10009055, Cayman Chemical). Concentration of lipid peroxides was calculated as an MDA equivalent using the extinction coefficient for the MDA–TBA complex of 1.56× 10^5^ M^−1^ cm^−1^ at 532 nm.

Total antioxidant capacity was assessed by using lag time by antioxidants against the myoglobin-induced oxidation of 2,2'-azino-di(3-ethylbenzthiazoline-6-sulfonic acid (ABTS) with H_2_O_2_ (709001, Cayman Chemical). Briefly, 20 μl of plasma samples (diluted 1:20, v/v) or heart homogenates (15 μg) were added in triplicate to 96-well plates, and mixed with 90 μl of 10 mM PBS (pH 7.2), 50 μl of myoglobin solution, and 20 μl of 3 mM ABTS. Reaction was initiated with H_2_O_2_ (20 μl) and change in color monitored at 600 nm (standard curve: 2–25 μM trolox).

To measure mitochondrial stress, cardiac myocytes (10^4^/well) were incubated with *Tc* and/or PJ34, and then loaded for 30 min with 5 μM MitoSOX red (detects mitochondrial ROS, Ex_498nm_/Em_598nm_) or 10 μM JC-1 (5,5′,6,6′-tetrachloro-1,1′,3,3′-tetraethylbenzimidazolylcarbocyanine iodide). Cells were washed, and JC-1 red aggregates (Ex_560nm_/Em_595nm_) vs. green monomers (Ex_485nm_/Em/_535nm_) ratio recorded by using a Spectra Max^R^ M2 microplate reader. The fluorescent probes were purchased from Invitrogen/Molecular Probes.

### Histology

Tissue sections were fixed in 10% buffered formalin, dehydrated in absolute ethanol, cleared in xylene, and embedded in paraffin. Five-micron tissue sections were subjected to Masson’s Trichrome staining at the Research Histopathology Core at the UTMB, and fibrosis was assessed by measuring the collagen area as a percentage of the total myocardial area (n = 4 mice /group, 2–3 slides per mouse, 10 microscopic fields per slide) using Simple PCI software (v.6.0; Compix, Sewickley PA). Sections were scored based on percent of fibrotic area: (0) <1%, (1) 1–5%, (2) 5–10%, (3) 10–15%, and (4) >15% [24].

### Echocardiography assessment of LV structure and function

Mice were continuously anesthetized by inhalant 1.5% isoflurane/100% O_2_ to maintain a light sedation level. Mice were placed supine on an electrical heating pad at 37°C during the examination. The echocardiography (ECG) electrodes of the Vevo 2100 ultrasound system (Visual Sonics, Toronto, Canada) were connected to mouse paws, and heart rate and respiratory physiology were continuously monitored. Mice chests were shaved, and warmed ultrasound gel was applied to the area of interest. Transthoracic echocardiography was performed using the high-frequency linear array transducer (MS400, 18–38 MHz) of the Vevo 2100 ultrasound system. Heart was imaged in B-mode and M-mode to examine the parameters of left ventricle (LV) in diastole (-d) and systole (-s). Pulse wave Doppler imaging was performed to measure heart diastolic function. All measurements were obtained in triplicate and data were analyzed by using Vevo 2100 standard measurement software [13].

### Data analysis

All data were analyzed by using a Prism5 (Graphpad, San Diego CA) or SPSS (IBM, Chicago IL) software, and expressed as mean ± standard error mean (SEM). Significance was calculated by using one variable comparison analyses (1-way ANOVA) with Tukey’s *post hoc* test, and multiple comparison analyses (2-way ANOVA) with Bonferroni post hoc test. If data were not normally distributed, then Mann-Whitney test and Kruskal-Wallis with Dunn’s test were employed. Significance is presented by * (WT.*Tc* vs. WT), ^&^ (PARP1^-/-^.*Tc* vs. PARP1^-/-^), and ^#^ (WT.*Tc* vs. PARP1^-/-^.*Tc*), and annotated as follows: *^,&,#^p<0.05, **^,&&,##^p<0.01, ***^,&&&,###^p<0.001).

## Results

### PARP1 and PARylation are increased in chagasic myocardium

**Fig 1:**
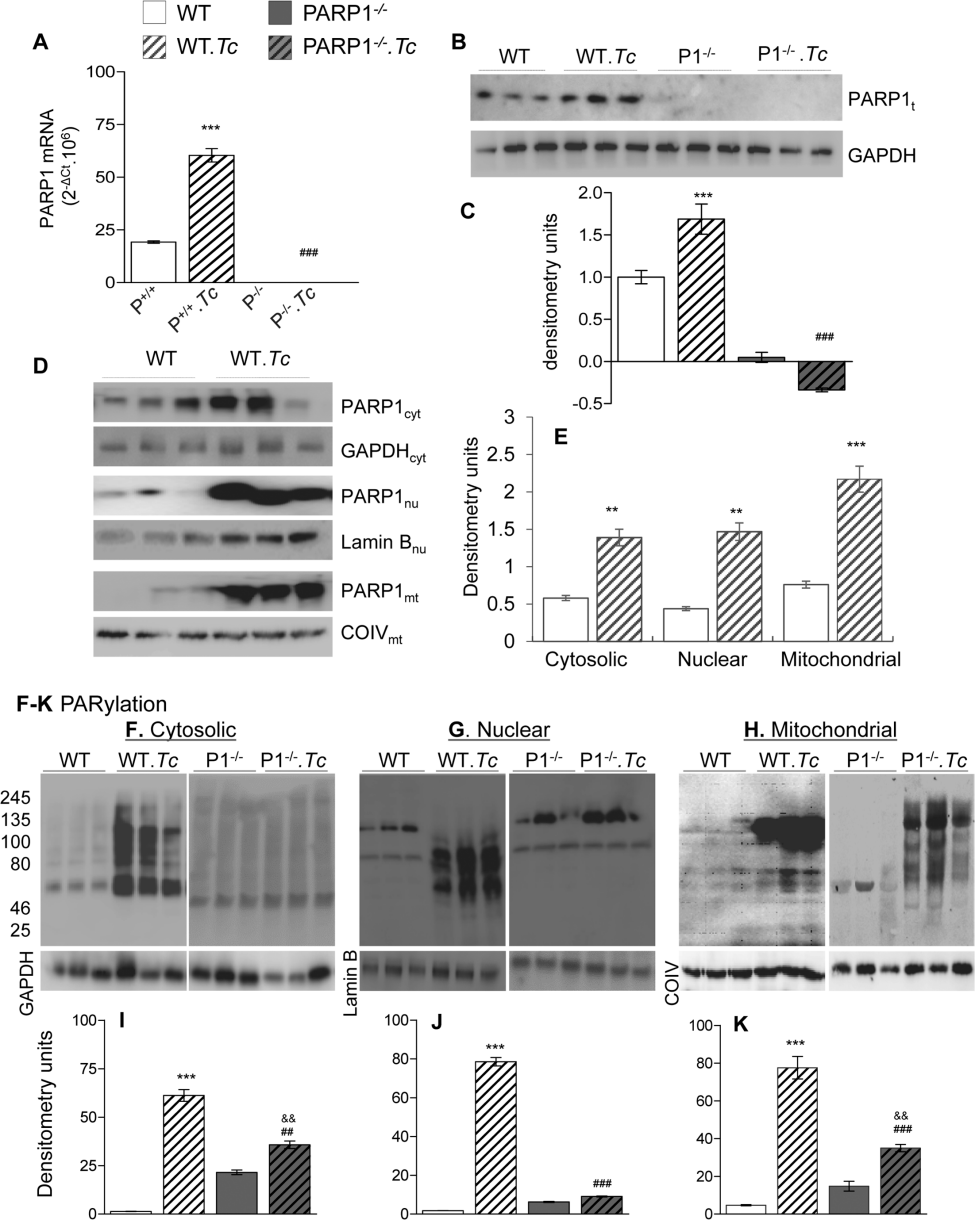
Myocardial PARP1/PAR status in chagasic WT and PARP1^-/-^ mice. C57/129SJ wild type (WT) and PARP1^-/-^ mice were infected with *Trypanosoma cruzi* (10,000 *Tc*/mouse), and sacrificed at 150 days’ post-infection (pi) corresponding to chronic disease phase. **(A)** RT-qPCR evaluation of myocardial levels of PARP1 mRNA, normalized to GAPDH mRNA (n ≥ 5 mice/group, triplicate observations per mouse). **(B-K)** Myocardial tissue homogenates and fractionated organelles were prepared as detailed in Materials and Methods. Representative Western blot images (n = 3 mice/group) are shown for total heart homogenate levels of PARP1 and GAPDH (**B)**, cytosolic levels of PARP1 and PAR with GAPDH as loading control **(D&F)**, nuclear levels of PARP1 and PAR with Lamin B as loading control **(D&G)**, and mitochondrial levels of PARP1 and PAR with COIV as loading control **(D&H)**. Densitometry analysis was performed for all Western blot gels from n ≥ 6 mice/group, and data for PARP1 and PAR levels in total heart homogenates and cytosolic fractions (**C,E,**&**I**), nuclear fractions (**E&J**) and mitochondrial fractions (**E&K**) are presented. In bar graphs, data are plotted as mean value ± SEM. Statistical significance is marked as *WT.*Tc* vs. WT, ^&^PARP1^-/-^.*Tc* vs. PARP1^-/-^, and ^#^WT.*Tc* vs. PARP1^-/-^.*Tc* (**^,&&,##^p<0.01, ***^,&&&,###^p<0.001).

The WT, PARP1^+/-^, and PARP1^-/-^ mice were genotyped to confirm the presence of two, one and no copies of the *PARP1* gene (S1A Fig). We then evaluated the effect of *Tc* infection on the levels of PARP1 mRNA, protein, and activity in WT and PARP1^-/-^ mice. In PARP1^-/-^ mice, PARP1 mRNA and protein levels remained undetectable before or after *Tc* infection (Fig 1A–1C). In chronically infected WT (vs. uninfected WT) mice, the myocardial levels of PARP1 mRNA and protein were increased by 220% and 68.8%, respectively (Fig 1A–1C, all, p<0.001). Tissue fractionation/Western blotting showed 130%, 230%, and 141% increase in PARP1 levels in the myocardial cytosolic, nuclear, and mitochondrial fractions of WT.*Tc* (vs. WT) mice (Fig 1D & 1E, all, p<0.01). Protein PARylation is an indicator of PARP1 activity. Our data showed 43-fold, 44-fold and 30-fold increase in protein PARylation levels, respectively, in the myocardial cytosolic, nuclear, and mitochondrial fractions of WT.*Tc* (vs. WT) mice (Fig 1F–1K, all, p<0.001). The PARP1^-/-^ mice exhibited a basal PARylation level that was not substantially increased in response to chronic infection (Fig 1F–1K). These results suggest that PARP1 expression and PARylation activity were increased in myocardial fractions (cytosolic, nuclear and mitochondrial) of chagasic WT mice. Other members of the PARP family likely contributed to the slight increase in PARylation level observed in chronically infected PARP1^-/-^ mice.

### PARP1/PAR role in mtDNA integrity (± *T*. *cruzi*)

**Fig 2:**
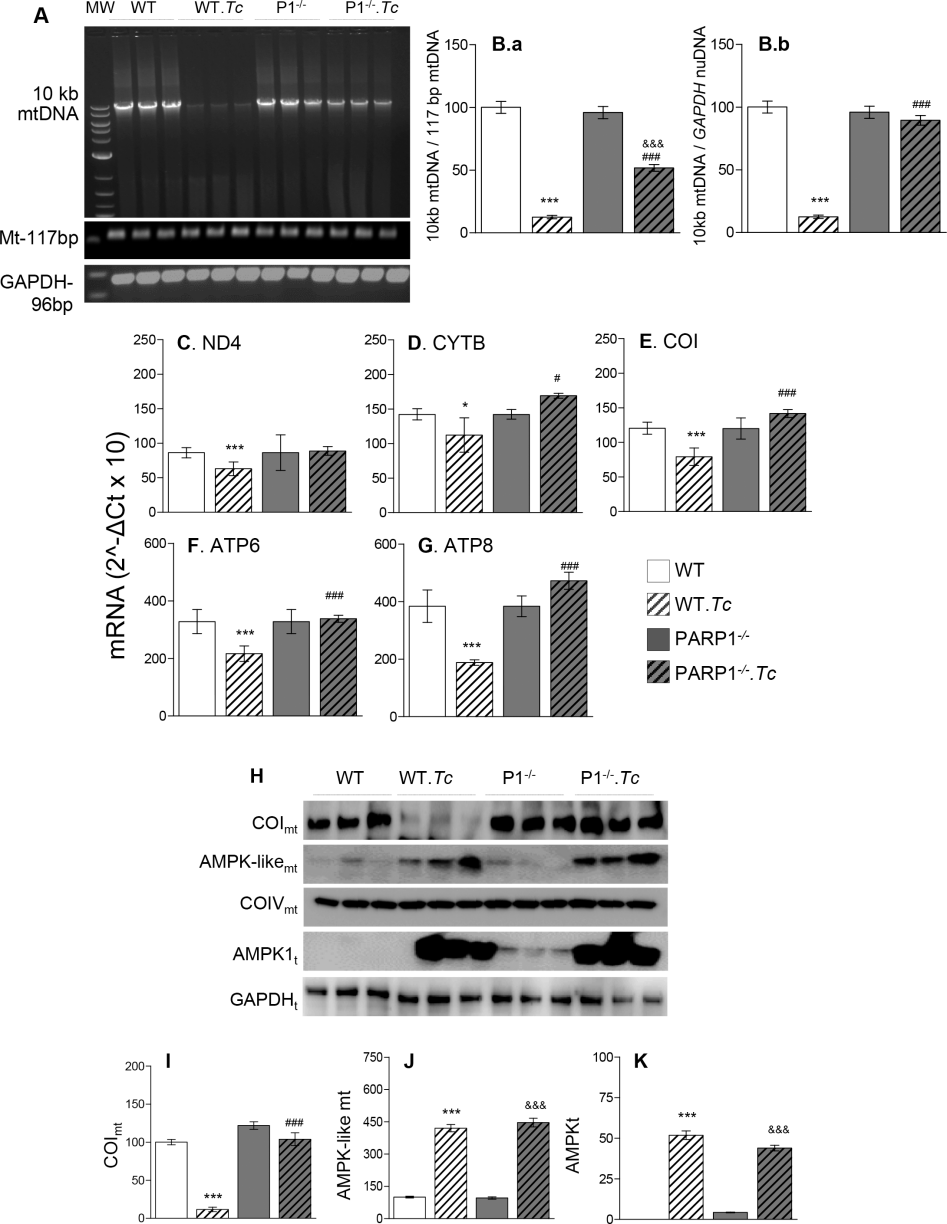
Mitochondrial DNA integrity and expression of mtDNA-encoded genes in chagasic mice (± PARP1). Mice (WT and PARP1^-/-^) were infected with *T*. *cruzi* and sacrificed at 150 days’ pi. **(A)** Representative gel images (n = 3 mice/group) show myocardial levels of 10 kb mtDNA with short 177-bp mtDNA and 96-bp *GAPDH* (nuDNA) fragments as controls. The PCR amplification was conducted for 28 cycles. **(B)** Densitometry analysis was performed on PCR gels representing n≥ 6 mice/group, and density of the 10 kb mtDNA band was normalized against mtDNA ***(B*.*a)*** and nuDNA ***(B*.*b)*** fragments. **(C-G)** Myocardial mRNA levels for mtDNA-encoded genes were determined by RT-qPCR, and normalized to *GAPDH* mRNA (n ≥ 5, triplicate observations per mouse). **(H-K)** Representative Western blotting images (n = 3 mice per group) of cardiac mitochondrial levels of COI and AMPK-like (COIV, loading control) and total heart homogenate levels of AMPK (GAPDH, loading control) are shown **(H)**. Densitometry analysis was performed on gel images for n = 6 mice for each protein, and data for COI and AMPK-like levels in mitochondrial fractions **(I&J)** and AMPK levels in total heart homogenates **(K)** were normalized to COIV and GAPDH, respectively. In bar graphs, data are plotted as mean value ± SEM. Statistical significance are marked as *WT.*Tc* vs. WT, ^&^PARP1^-/-^.*Tc* vs. PARP1^-/-^, and ^#^ WT.*Tc* vs. PARP1^-/-^.*Tc* (*^,#^p<0.05, ***^,&&&,###^p<0.001).

Because PARP1 is a DNA repair enzyme and mtDNA encodes essential components of the respiratory chain, we determined if mitochondrial translocation of PARP1/PAR preserved the mtDNA in chagasic myocardium. Long qPCR amplification of 10 kb mtDNA fragment showed the basal level of mtDNA content was not changed in WT, PARP1^+/-^, and PARP1^-/-^ mice. The long mtDNA (vs. short mtDNA fragment) in chagasic WT, PARP1^+/-^, and PARP1^-/-^ mice (vs. matched controls) was decreased by 70%, 46%, and 32%, respectively (Fig 2A, Fig 2B.a, and S2 Fig panels A & B.a, p<0.001). When compared to nuDNA fragment, the long mtDNA content was decreased by 85% and 30%, respectively, in the myocardium of chagasic WT and PARP1^+/-^ mice (p<0.001) and no change was noted in PARP1^-/-^ mice *(*Fig 2B.b, S2 Fig panel B.b). The protection of mtDNA content was associated with preservation of mtDNA-encoded genes’ expression at mRNA (*ND4*, *CYTB*, *COI*, *ATP6*, *and ATP8*, Fig 2C–2G) and protein (COI, Fig 2H & 2I) levels in chronically infected PARP1^-/-^ (vs. control) mice. In comparison, WT.*Tc* (vs. WT) mice exhibited 21–51% decline in mtDNA-encoded gene expression and >80% decline in COI protein level (Fig 2C–2I, p<0.05). Mitochondrial (and total) levels of AMPK-like protein were equally increased in chagasic WT and PARP1^-/-^ mice, and no changes in COIV (nuDNA encoded complex IV subunit) were noted in WT and PARP1^-/-^ mice (Fig 2H, 2J & 2K). Our results suggest that a) *Tc*-induced PARP1/PAR were detrimental to mtDNA integrity and mtDNA-encoded gene expression, and b) depletion of PARP1/PAR prevented the loss in mtDNA content in chronic Chagas disease. Though a potential contamination with cytoplasmic material may explain the finding of AMPK-like protein in mitochondrial fractions, we believe that this observation indicates a novel role for AMPK in mitochondrial biogenesis.

### PARP1/PAR binding to Pol γ replisome in *T*. *cruzi* infection

**Fig 3:**
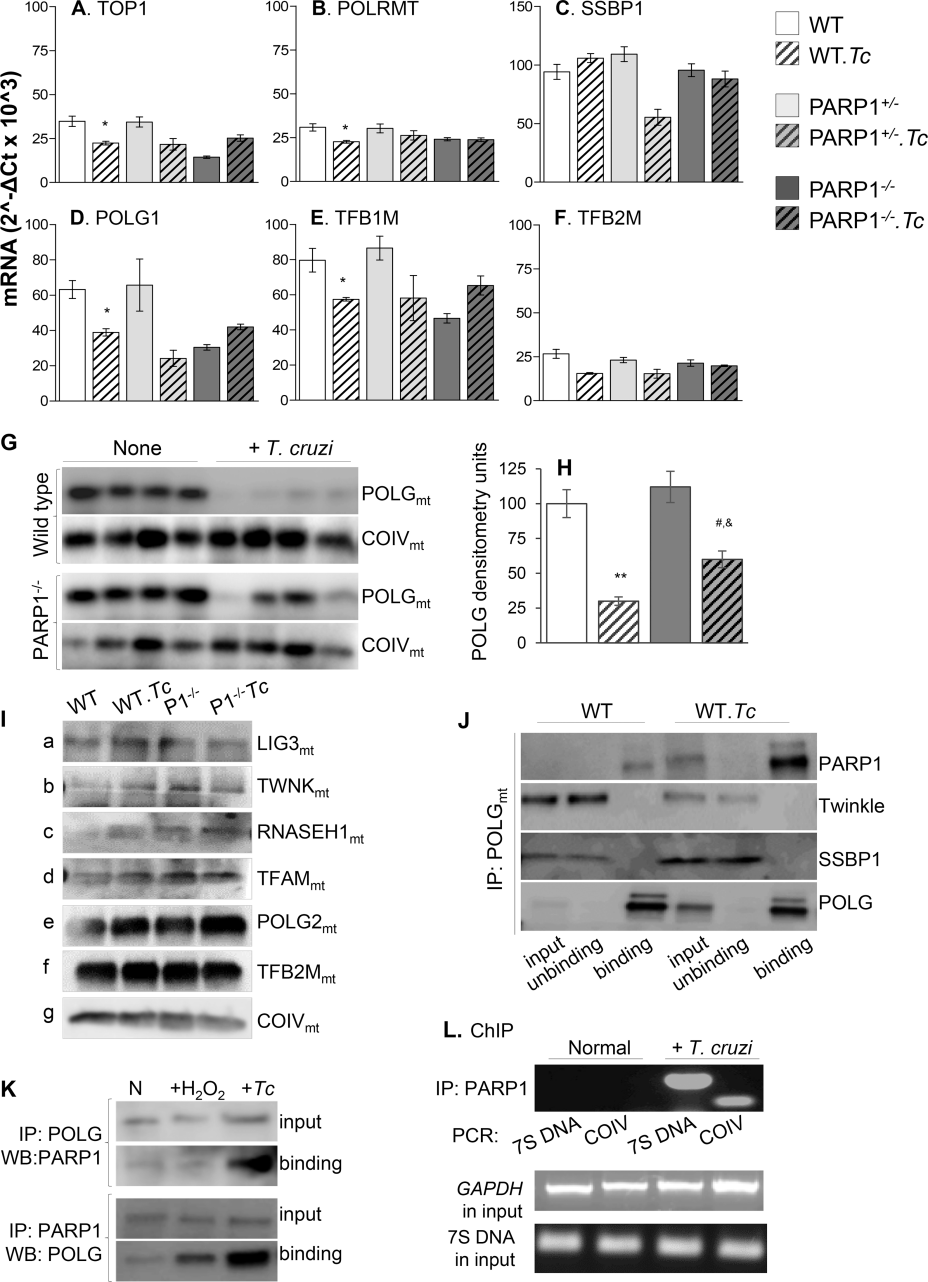
Expression of mtDNA replication machinery and POLG interaction with PARP1 in chagasic mice. **(A)** Mice (WT, PARP1^+/-^, and PARP1^-/-^) were infected with *Tc* and sacrificed at 150 days’ pi. **(A-F)** Shown is RT-qPCR evaluation of myocardial levels of mRNAs for components of the mtDNA replication and transcription machinery in chronically infected and control mice. Data were normalized to GAPDH mRNA (n ≥ 5 mice/group, triplicate observations per mouse). **(G-I)** Heart homogenates were subjected to differential centrifugation to isolate mitochondria. Representative Western blot images for cardiac mitochondrial levels of POLG (***G***, n = 4 mice/group) and other proteins of mtDNA replication machinery (**I**) in WT and PARP1^-/-^ mice are shown (loading control: COIV). Densitometry analysis was performed for Western blot gels representing n≥ 4 mice/group, and data were normalized to COIV levels (***H***). **(J)** Cardiac mitochondria from normal and chagasic WT mice (n = 5/group) were pooled and subjected to immunoprecipitation with anti-POLG antibody. Input (total), flow-through (unbinding), and POLG-binding fractions were used for Western blotting with antibodies against PARP1, Twinkle, and SSBP1. WB with anti-POLG antibody was performed to confirm high efficiency of POLG pull down by immunoprecipitation in all samples. The gel images are representative of three independent experiments. **(K&L) PARP1-POLG binding and POLG activity in *Tc*-infected cells.** HeLa cells were infected with *T*. *cruzi* (cell: parasite ratio, 1:5) for 24 h (controls: uninfected or treated with 50 μM H_2_O_2_). Cells were used for cross-IP/WB with antibodies against POLG and PARP1 **(*K*)**. HeLa cells (± *T*. *cruzi*) were treated with formaldehyde to cross-link proteins and DNA. After immunoprecipitation with PARP1 antibody, DNA was used for PCR amplification of mitochondrial 7SDNA and nuclear COIV DNA fragments. The *GAPDH* and *7S* DNA in input samples were amplified to confirm equal loading of all samples **(*L*)**. Data in K and L are representative of three biological replicates. Data in bar graphs are plotted as mean value ± SEM, and statistical significance are marked as *WT.*Tc* vs. WT, ^&^PARP1^-/-^.*Tc* vs. PARP1^-/-^, and ^#^WT.*Tc* vs. PARP1^-/-^.*Tc* (*^,&,#^p<0.05, **p<0.01).

Because mtDNA content was improved in PARP1^-/-^ (vs. WT) chagasic mice, we considered that PARP1/PAR constitute a risk factor by adversely affecting the mtDNA replication. The mRNA levels for the components of the mtDNA replication/repair complex, including TOP1mt, POLRMT, and SSPB1, as well as the genes of the mitochondrial transcription machinery including TFB1M and TFB2M were slightly decreased or not changed in the myocardium of WT, PARP1^+/-^ and PARP1^-/-^ mice chronically infected with *T*. *cruzi* (vs. matched controls, Fig 3A–3F). Only POLG exhibited 35% decline at mRNA level (Fig 3D) and a significant decline at mitochondrial protein level in the myocardium of WT.*Tc* (vs. WT) mice (Fig 3G & 3H, p<0.05). The POLG mRNA level was preserved to normal level and mitochondrial POLG protein level was substantially preserved in PARP1^-/-^.*Tc* (vs. WT.*Tc)* mice (Fig 3D, 3G & 3H). No significant decline in the mitochondrial levels of other proteins of the mtDNA biogenesis machinery, including LIG3, TWNK, RNASEH1, TFAM, POLG2, and TFB2M was observed in cardiac mitochondria of chronically infected WT and PARP1^-/-^ (vs. matched controls) mice (Fig 3I & 3J). Immunoprecipitation/Western blotting (IP/WB) showed that the direct binding of POLG to PARP1 (but not to TWNK and SSBP1) was increased in cardiac mitochondria of chagasic WT mice (Fig 3K). The POLG was pulled down with high efficiency as is evidenced by WB finding of all of the POLG in binding fraction and none in unbinding fractions (Fig 3K, bottom panel). We also monitored PARP1/POLG interaction in HeLa cells infected with *T*. *cruzi*. Cross-IP/WB showed that POLG pull-down of PARP1 (and vice versa) was increased in mitochondria of infected (vs. normal) HeLa cells (Fig 3L). Treatment of HeLa cells with 50 μM H_2_O_2_ did not enhance POLG/PARP1 interaction to the same extent as was noted with *Tc* infection (Fig 3L). Chromatin immunoprecipitation with anti-PARP1 antibody followed by PCR showed that PARP1 binding to mtDNA sequence (7S near D-loop) and nuclear sequence (COIV) was also increased in infected HeLa cells (Fig 3M). No changes in input *7S* mtDNA and *GAPDH* nuDNA were noted (Fig 3M, bottom panels). Together, the results presented in Fig 3, along with those presented in Fig 2, suggest that a) nuclear PARP1 does not affect the expression of a majority of the components of the mtDNA replication machinery in chagasic myocardium. However, b) mitochondrial PARP1 binding to POLG and mtDNA was increased in the myocardium of chronically infected mice, and c) genetic depletion of PARP1 was beneficial in preserving the POLG-dependent mtDNA content in cardiac mitochondria of chagasic mice.

### PARP1/PAR effects on mitochondrial function and antioxidant/oxidant balance

**Fig 4:**
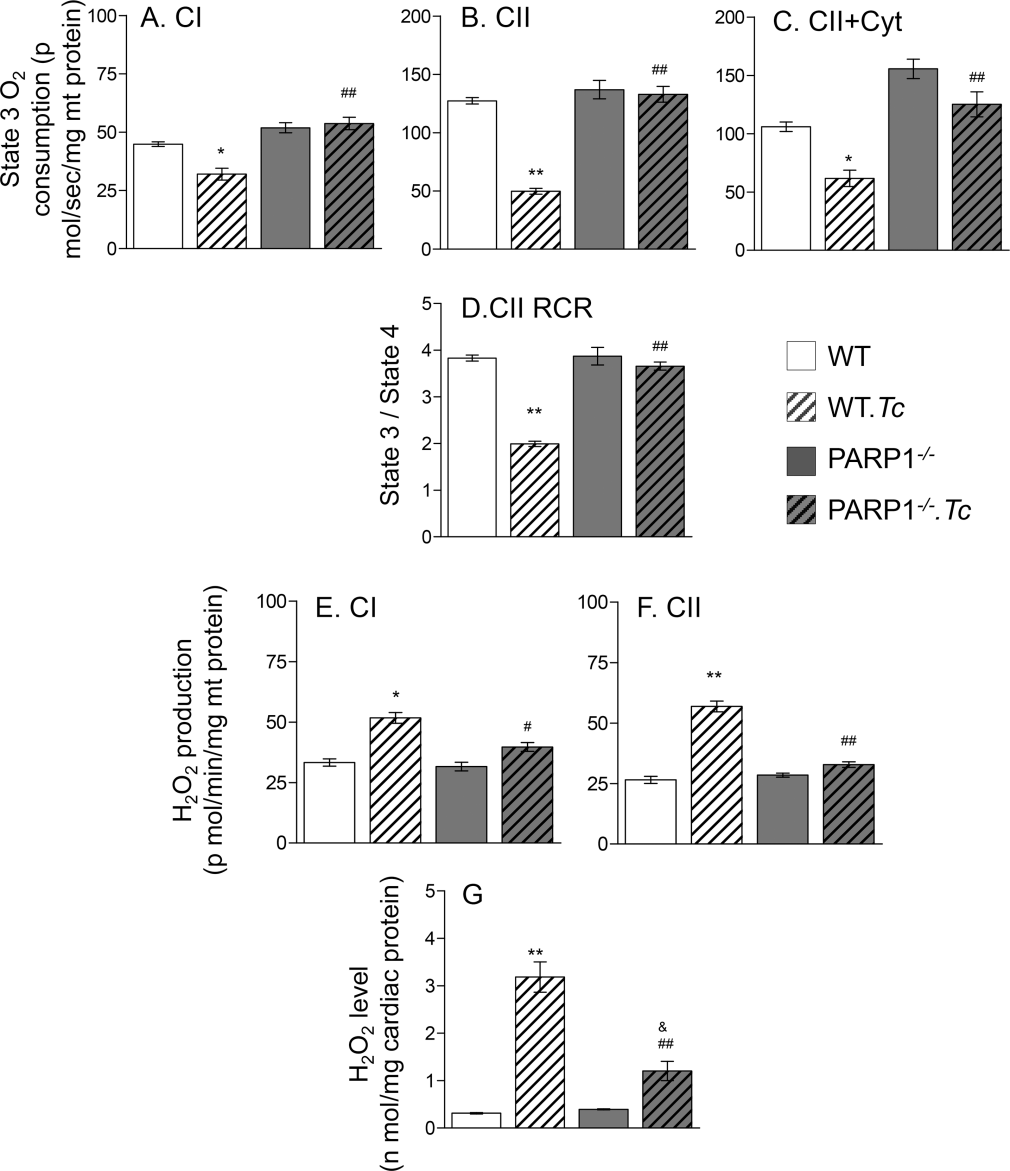
Mitochondrial respiration and ROS production in chronically infected WT and PARP1^-/-^ mice. WT and PARP1^-/-^ mice were harvested at 150 days’ post-infection. **(A-D)** Cardiac myofiber bundles (~2 mg) were permeabilized, and mitochondrial respiratory function was measured using an Oxygraph-2k respirometer. After recording the state 4 respiration with addition of CI substrates (glutamate/pyruvate/malate), ADP was added, and state 3 respiration with electron input from complex CI was recorded (***A***). Rotenone (inhibits complex I) and CII substrate (succinate) were added to record CII-supported state 3 respiration (***B***). Competence of the outer mitochondrial membrane was assessed with addition of cytochrome c (***C***). The CII driven respiratory control ratio (RCR) is presented in ***D***. **(E-G)** Isolated cardiac mitochondria were incubated in presence of CI (**E**) and CII (**F**) substrates, and the rate of H_2_O_2_ production was recorded. The total H_2_O_2_ levels in heart homogenates is shown in ***G***. Data in all bar graphs are plotted as mean value ± SEM (n ≥ 5 mice/group, triplicate observations per sample). Statistical significance are marked as *WT.*Tc* vs. WT, ^&^PARP1^-/-^.*Tc* vs. PARP1^-/-^, and ^#^WT.*Tc* vs. PARP1^-/-^.*Tc* (*^,&,#^p<0.05, **^,##^p<0.01).

The mtDNA encodes for essential components of respiratory complexes and its deficiency can directly disturb the mitochondrial function. We, therefore, monitored if PARP1/PAR affect the OXPHOS capacity in chagasic myocardium. No significant differences in the basal level of mitochondrial respiration and state 4 respiration driven by CI or CII substrates were observed in the myocardial fibers of WT and PARP1^-/-^ mice in presence or absence of chronic *Tc* infection. The CI and CII substrates driven ADP-coupled state 3 respiration (indicates proton gradient for ATP synthesis) as well as respiratory control ratio (RCR, state 3 / state 4) were maintained to normal levels in chronically infected PARP1^-/-^ mice (Fig 4A, 4B and 4D). In comparison, we noted a 29% and 61% decline in CI- and CII-energized state 3 respiration, respectively (Fig 4A & 4B), and a 48% decline in CII-supported RCR (Fig 4D, all, p<0.05) in the myocardial fibers of WT.*Tc* (vs. WT) mice. Addition of cytochrome c did not improve the CII driven state 3 respiration in myocardial fibers of WT.*Tc* mice, thus, confirming that mitochondrial membranes were not damaged during experimental procedure (Fig 4C).

Mitochondrial respiratory dysfunction can result in increased release of electrons to molecular O_2_ and O_2_^●^ formation. Next, we determined if PARP1 depletion arrested the mtROS generation in chagasic heart. Isolated cardiac mitochondria of WT.*Tc* (vs. WT) mice exhibited 55% and 114% increase in CI- and CII-dependent H_2_O_2_ release, respectively, and myocardial H_2_O_2_ level was increased by >7-fold in chagasic WT mice (Fig 4E–4G, all, p<0.05). The PARP1^-/-^ mice exhibited non-significant changes in *Tc*-induced mtROS release, and only a modest increase in myocardial H_2_O_2_ level (Fig 4G). Together, the results presented in Fig 4, along with those presented in Fig 2 and Fig 3, suggest that a) PARP1 effects on mtDNA content contributed to a decline in mitochondrial OXPHOS capacity and an increase in mtROS production, and b) PARP1 depletion was beneficial in preserving the mitochondrial health in chagasic myocardium.

**Fig 5:**
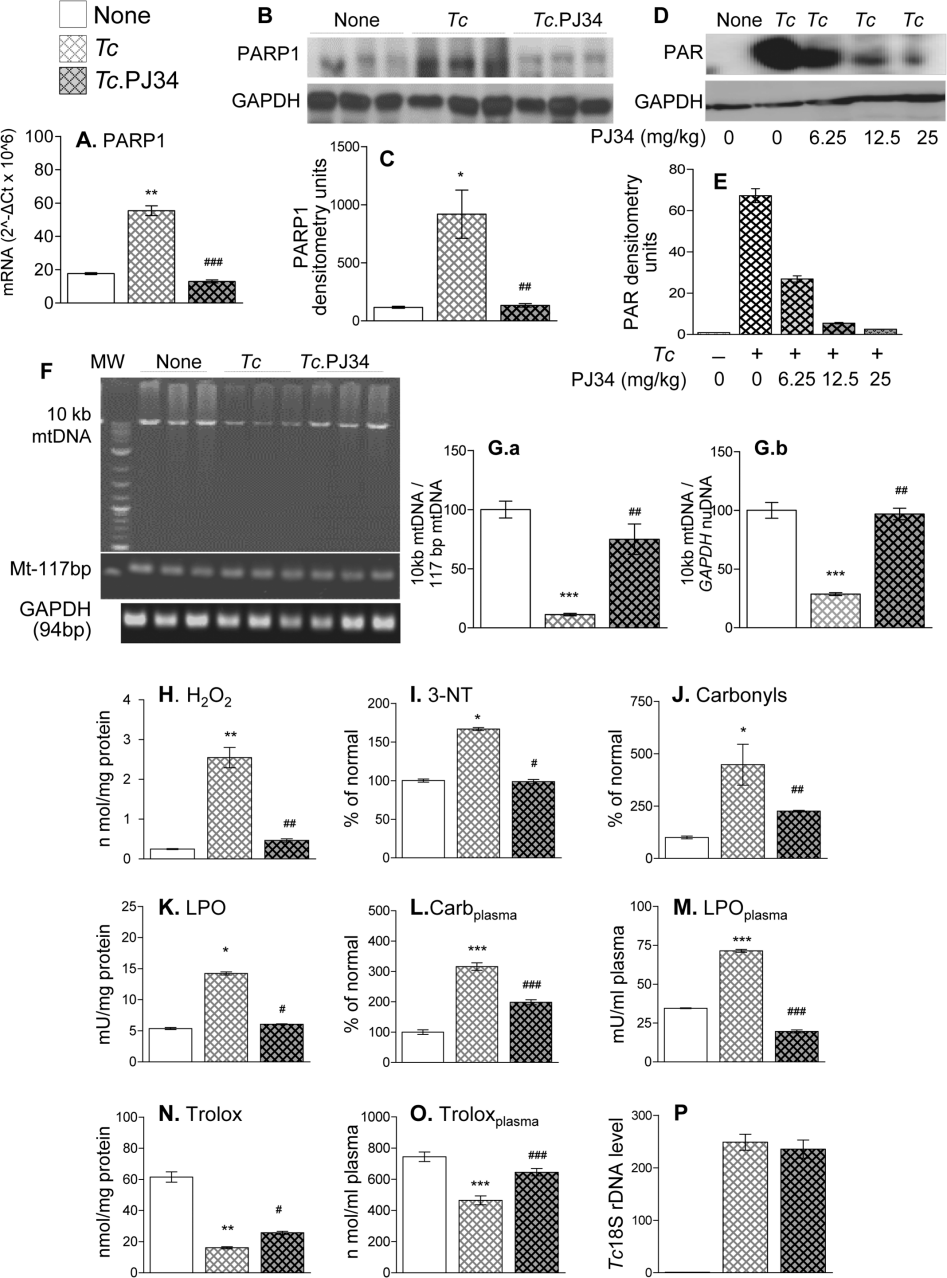
Treatment with PARP1 inhibitor improved mitochondrial biogenesis in chagasic mice. C57BL/6 mice were infected with *T*. *cruzi*, treated with PJ34 (12.5 mg/100-μl/mouse, intraperitoneally, twice a week for three weeks beginning at 45 days’ pi, and sacrificed at 150 days’ pi. **(A)** RT-qPCR evaluation of myocardial level of PARP1 mRNA, normalized to GAPDH mRNA (n ≥ 5 mice/group, triplicate observations per mouse). **(B-E)** Representative Western blot images of myocardial level of PARP1 with GAPDH loading control (n = 3 mice/group) and PAR levels in mice treated with increasing concentration of PJ34 (0–25 mg/kg) are shown in ***B*** & ***D***, respectively. Densitometry analysis was performed for all Western blot gels from n ≥ 6 mice/group, and PARP1 and PAR levels (normalized to GAPDH levels) are shown in ***C*** & ***E***, respectively. **(F&G)** Representative gel images **(F**, n = 3 mice/group) show myocardial levels of 10 kb mtDNA with 177-bp mtDNA and 96-bp *GAPDH* (nuDNA) fragments as controls. The PCR amplification was performed for 28 cycles. Densitometry analysis was performed on PCR gels representing n ≥ 6 mice/group, and density of the 10 kb mtDNA band, normalized against mtDNA and nuDNA fragments, is presented (***G*.*a*&*b***). **(H-O)** Bar graphs (n ≥ 5 mice/group, duplicate or triplicate observations per sample) show the myocardial (**H-K & N**) and plasma (**L, M & O**) levels of H_2_O_2_ **(*H*)**, 3-nitrotyrosine **(*I*)**, protein carbonyls **(*J*&*L*)**, lipid hydroperoxides **(*K*&*M*)** and antioxidant capacity **(*N*&*O*)**. (**P**) Myocardial parasite burden in chronically infected (± PJ34 treatment) mice was determined by qPCR amplification of *Tc*18SrDNA and normalized with GAPDH (n≥ 5 mice/group, three observations per mouse). Data in all bar graphs are plotted as mean value ± SEM, and statistical significance are marked as *infected vs. control and ^#^infected/PJ34-treated vs. infected/untreated (*^,#^p<0.05, **^,##^p<0.01, ***^,###^p<0.01).

We confirmed the effects of PARP1/PAR on mtDNA content and antioxidant/oxidant balance by using a small molecule inhibitor of PARP1. Mice were infected with *T*. *cruzi* and then treated with a selective PARP1 inhibitor (PJ34, 12.5 mg/kg) as described in Materials and Methods. The PJ34 treatment abolished the >2-fold increase in PARP1 mRNA and protein levels observed in the myocardium of chagasic mice (Fig 5A–5C, p<0.001), as has also been observed previously in brain endothelial cells [25]. The *Tc*-induced increase in PARylation level, a measure of PARP1 activity, was also controlled by PJ34 in a dose dependent manner (Fig 5D & 5E). Further, PJ34 treatment resulted in >90% recovery of the mtDNA content (Fig 5F & 5G.a&b), 54–100% control of myocardial (Fig 5H–5K) and plasma (Fig 5L & 5M) levels of H_2_O_2_, 3-nitrotyrosine, protein carbonyls, and lipid hydroperoxides, and 21–64% recovery of the myocardial and plasma antioxidant capacity (Fig 5N & 5O) in chagasic mice. No effects of PJ34 were noted on the parasite load in chagasic/treated (vs. chagasic/untreated) mice (Fig 5P). Similar to the findings in mice, treatment of cardiac myocytes with PJ34 controlled the *T*. *cruzi* induced increase in the levels of PARP1 mRNA (S2C Fig) and cellular and mitochondrial oxidative stress (S2I and S2J Fig), and the decline in the expression of the components of the mtDNA replication machinery (S2D–S2H Fig) and mitochondrial health measured by the JC1 red/green ratio (S2K Fig). Together, these results suggest that PJ34-dependent control of PARP1/PAR was effective in preserving the mtDNA content, mitochondrial health, and antioxidant/oxidant balance that otherwise were profoundly disturbed in the cardiomyocytes and murine heart by *T*. *cruzi* infection.

### PARP1/PAR depletion improved the cardiac structure and LV function in chagasic mice

**Fig 6:**
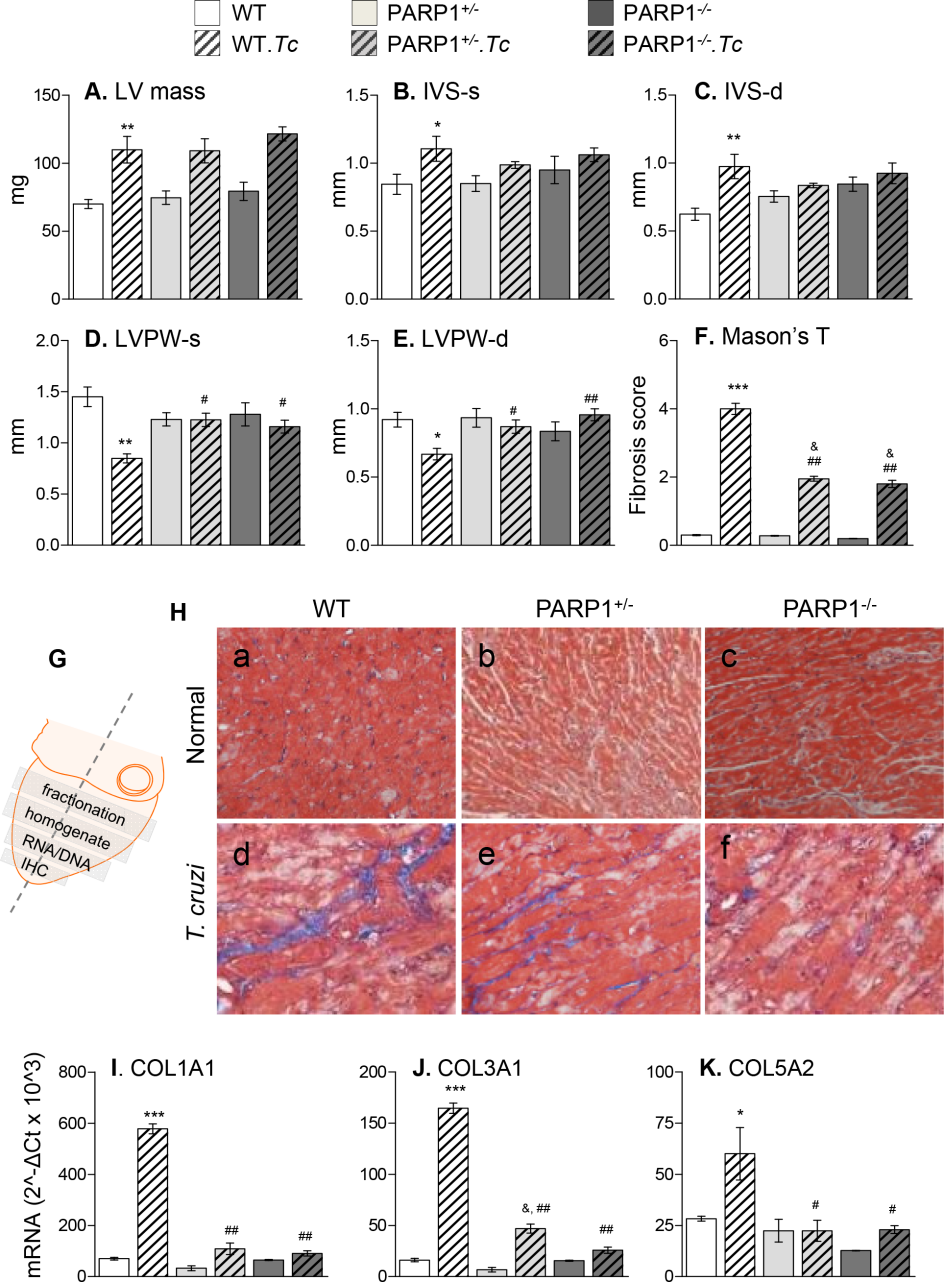
Cardiac remodeling in chagasic mice (± PARP1). Mice (WT, PARP1^+/-^, and PARP1^-/-^) were infected with *T*. *cruzi*, and sacrificed at 150 days’ pi. **(A-E)** Cardiac structural changes were analyzed by echocardiography using a Vevo 2100 System. Shown are left ventricular (LV) mass **(*A*)**, and systolic (-s) and diastolic (-d) changes in the thickness of the interventricular septum **(**IVS, ***B*&*C*)** and LV posterior wall **(**LVPW, ***D*&*E*)** in chronically infected and matched control mice (n = 8–12 mice/group, triplicate recordings per mouse). **(F-H)** Hearts were sectioned for various experiments as shown in **G**. Apex heart sections were stained with Mason’s trichrome, and representative images are shown in ***H*.*a-f***. Tissue sections were scored for collagen (***F***, n = 4 mice/group, 2 slides per mouse, 10 microscopic fields per slide) as described in Materials and Methods. **(I-K)** Real time RT-qPCR analysis of myocardial levels of mRNAs for collagen isoforms COLI, COLIII, and COLV, in chronically infected (and control) WT, PARP1^+/-^, and PARP1^-/-^ mice (n ≥ 5 mice/group, triplicate observations per mouse). Data were normalized to *GAPDH* mRNA. Data in all bar graphs are plotted as mean value ± SEM, and statistical significance are marked as *WT.*Tc* vs. WT, ^&^genetically modified/infected vs. matched controls, and ^#^WT.*Tc* vs. genetically-modified/infected (*^,&,#^p<0.05, **^,##^p<0.01, ***p<0.001).

ROS can signal fibrosis, and chronic hypertrophy is a key cause for LV dysfunction. We, therefore, examined if control of PARP1-dependent mitochondrial impairment and oxidative stress arrested cardiac remodeling and LV dysfunction in Chagas disease. Echocardiography imaging showed the LV mass and inter-ventricular septum thickness (IVS) were increased by 29–58%, while LV posterior wall (LVPW) was thinned by 31–47% in chagasic (vs. control) WT mice (Fig 6A–6E, all, p<0.05, S2 Table). Histological evaluation of tissue sections by Masson's Trichrome staining showed the myocardial collagen content was significantly increased in chagasic myocardium (score: 4.0 ± 0.4 vs. 0.3 ± 0.04, WT.*Tc* vs. WT, Fig 6F–6H, p<0.001). An increase in cardiac fibrosis in WT.*Tc* (vs. control) mice was also evidenced by 6-fold, 8-fold, and 1.5-fold increase in mRNA levels for COL1A1, COL3A1, and COL5A2, respectively (Fig 6I–6K, all, p<0.05). The chronically infected PARP1^+/-^ and PARP1^-/-^ mice exhibited the ability to maintain IVS and LVPW thickness at normal levels, >50% decline in collagen deposition, and 50–90% decline in the mRNA levels of collagen isoforms when compared to that noted in WT.*Tc* mice (Fig 6A–6K, all, p<0.05).

**Fig 7:**
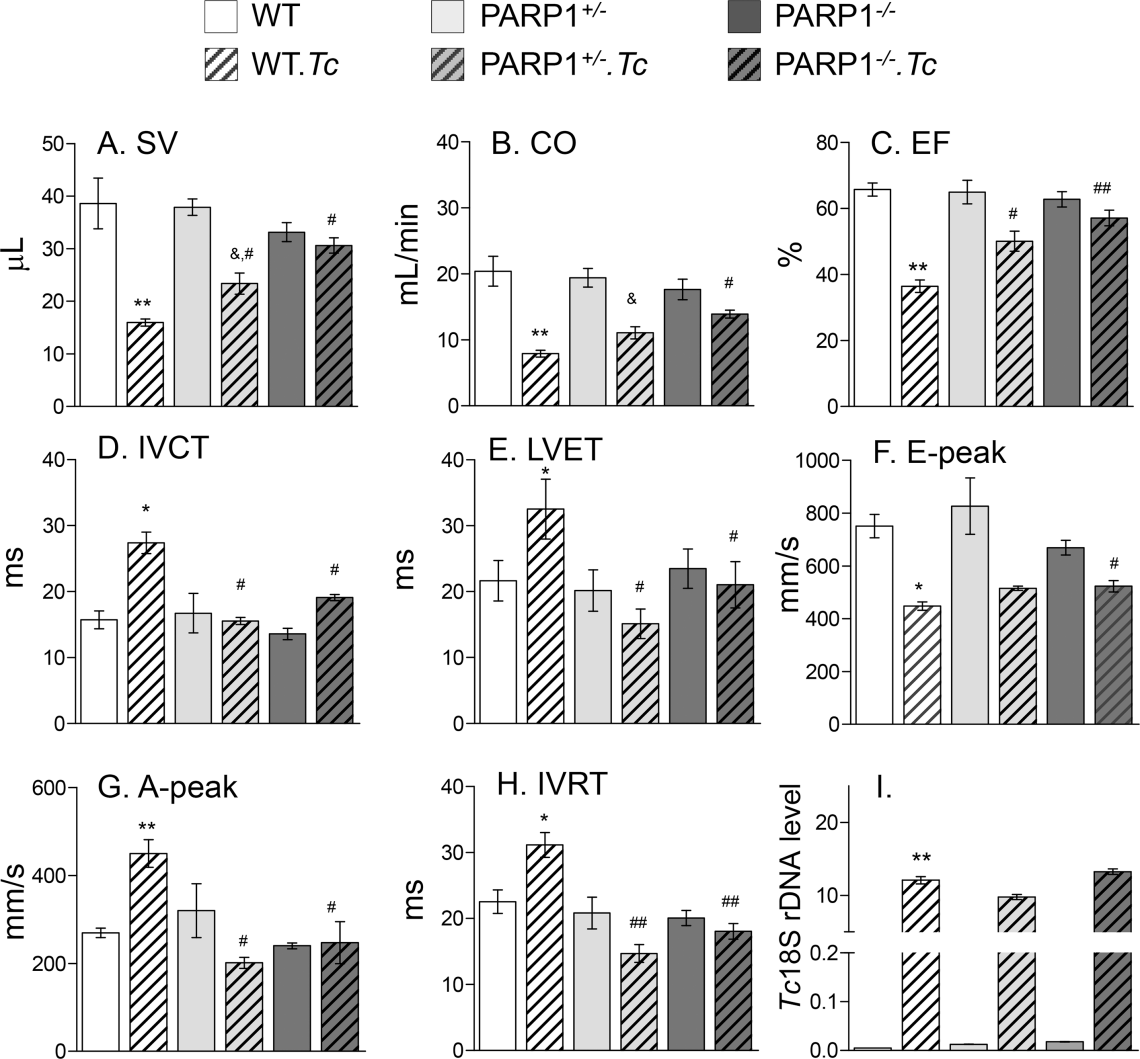
Left ventricular systolic and diastolic performance in chagasic mice (± PARP1). WT, PARP1^+/-^, and PARP1^-/-^ mice were monitored at 150 days’ post-infection. Shown are transthoracic echocardiography measurements of **(A)** stroke volume (SV), **(B)** cardiac output (CO), and **(C)** ejection fraction (EF). Pulse-wave doppler echocardiography was performed to measure **(D)** isovolumic contraction time (IVCT), **(E)** LV ejection time, mitral valve **(F)** early and **(G)** late peak velocities, and **(H)** isovolumic relaxation time (IVRT). The data presented in ***A-H*** were acquired from n = 8–12 mice/group with triplicate recordings per mouse. **(I)** Myocardial parasite burden was determined by qPCR amplification of *Tc*18SrDNA and normalized with *GAPDH* (n≥ 5 mice/group, three observations per mouse). Data in all bar graphs are plotted as mean value ± SEM. Statistical significance are marked as *WT.*Tc* vs. WT, ^&^genetically modified/infected vs. matched controls, and ^#^WT.*Tc* vs. genetically-modified/infected (*^,&,#^p<0.05, **^,##^p<0.01). Detailed LV function data are presented in S2 Table.

The indices of LV systolic function, i.e., stroke volume (SV), cardiac output (CO), and ejection fraction (EF), were decreased by 66%, 51% and 46% respectively, in WT.*Tc* (vs. WT) mice (Fig 7A–7C, all, p<0.01). The systolic dysfunction prolonged the pre-ejection isovolumic contraction time (IVCT, 74% increase, Fig 7D, p<0.05) and shortened the LV ejection time (LVET, Fig 7E, p<0.05) in chagasic mice. Further, 40–67% changes in the early (E) and late (A) diastolic filling velocities indicated diastolic dysfunction in chagasic WT mice (Fig 7F & 7G, all, p<0.05). Both systolic and diastolic dysfunction contribute to abnormality in myocardial relaxation that was presented by 50% increase in isovolumic relaxation time (IVRT, Fig 7H, p<0.05) in WT.*Tc* mice. In comparison to WT.*Tc* mice, PARP1^+/-^.*Tc* and PARP1^-/-^.*Tc* mice exhibited a partial-to-full control of systolic and diastolic dysfunction, and myocardial contraction and relaxation indices, the maximal benefits of PARP1 deletion being observed in PARP1^-/-^ mice (Fig 7A–7H).

Finally, we obtained a quantitative measure of tissue parasite burden to confirm if the observed benefits of PARP1 depletion are delivered through its effects on parasite persistence. Myocardial level of *Tc*18SrDNA were similarly increased in chagasic WT, PARP1^+/-^, and PARP1^-/-^ mice (Fig 7I). Together, the results presented in Fig 6 and Fig 7 suggest that changes in the LV walls’ thickness (and thereby contractile capacity) contributed to compromised systolic and diastolic performance of the heart in chagasic WT mice. The benefits of PARP1 depletion in preserving LV hemodynamics and myocardial performance were delivered via control of collagenosis and stiffness of IVS and LVPW in the myocardium of chagasic mice.

## Discussion

PARP1 utilizes NAD^+^ as substrate and attaches ADP-ribose units to amino acids on various acceptor proteins. The nuclear PARP1/PAR was discovered more than fifty years [26]. In the nucleus, PARP1 activity is mainly targeted towards PARylation of lysine, arginine, and cysteine residues, and it participates in nuclear DNA repair through PARylation of histone proteins [17]. Recent studies show that major histone proteins, H2A and H2B involved in nuclear DNA repair, are also present in the inner mitochondrial membranes [27], and intra-mitochondrial localization of PARP1 and PARP1 activity were increased in cultured cells exposed to genotoxic stimuli (e.g. ROS) [28]. However, investigators have largely ignored studying the role of mtPARP1/PAR in health and disease. In this study, we have found that the expression, activity, and intra-mitochondrial localization of PARP1/PAR were increased in cardiac biopsies of chagasic mice and in human cardiomyocytes infected by *Tc*. While the expression of a majority of the components of the mtDNA replisome machinery was not significantly altered in chagasic (vs. control) mice, PARP1 binding to POLG and mtDNA was increased and associated with a loss in mtDNA content, mtDNA-encoded gene expression, and OXPHOS capacity, and an increase in mitochondrial ROS production in chronically infected murine myocardium. Importantly, Inhibition of PARP1 by genetic deletion or treatment with a chemical antagonist had protective effects against cardiac hypertrophy and LV dysfunction in chagasic heart. The beneficial effects of PARP1 inhibition were not delivered via its direct effect on parasite persistence that is suggested to be the major cause for the development of chronic cardiomyopathy (reviewed in [29,30]). Instead, PARP1 depletion preserved the POLG-dependent mtDNA content, mitochondrial function, and antioxidant/oxidant balance in chagasic myocardium and human cardiomyocytes infected by *T*. *cruzi*. Taken together, we have demonstrated that the mitochondrial transport of PARP1/PAR adversely impacts the mtDNA maintenance by POLG replisome, and exacerbates the mitochondrial dysfunction, oxidative stress and cardiac remodeling in Chagas disease. We propose that PARP1 inhibitors will be beneficial in preserving the mitochondrial health and LV function in chronic cardiomyopathy of chagasic (and potentially other) etiologies.

It is logical to expect that mitochondrial PARP1, via its capacity to carry out PARylation, can modulate mitochondrial function. A proteomic study has indeed reported an increase in PARylation of a variety of mitochondrial proteins in response to traumatic brain injury in rodents [31], and Modis et al [32] have found mitochondrial respiratory deficiency in A549 human epithelial cells overexpressing mitochondria targeted PARP1. In our *in vivo* model of Chagas disease, cytosolic, nuclear, as well as mitochondrial PARP/PAR were increased in the myocardium of infected mice. The organelle-specific deletion or overexpression of PARP1 *in vivo* is not yet feasible, and thus it is difficult to dissect the role of nuclear (vs. mitochondrial) PARP1/PAR in health and disease. Nevertheless, we noted that the nuclear-DNA encoded proteins of the respiratory complexes as well as majority of the proteins of POLG replisome were not altered in expression in chagasic (vs. control) heart. This observation suggests that nuclear PARP1/PAR is either beneficial or not detrimental in Chagas disease. Instead, the increase in intra-mitochondrial PARP1/PAR was associated with mitochondrial electron transport chain dysfunction, and decreased the CII-complex supported ATP synthesis. Further, the dysregulation of mitochondrial electron transport chain resulted in mitochondrial uncoupling, and produced a secondary increase in ROS by mitochondria. PARP1/PAR inhibition, in a dose dependent manner, was beneficial in rescuing the mitochondrial electron transport chain activity and OXPHOS capacity in chagasic heart. Sirtuin 1 is a highly conserved member of the family of NAD^+^-dependent Sir2 histone deacetylases [33], and it competes with PARP1 for NAD^+^ substrate. Bai et al showed that PARP1 or PARP2 deficiency enhanced the mitochondrial oxidative metabolism, energy expenditure, and protection against diet-induced obesity, and this outcome was achieved, at least partially, via increased availability of NAD^+^ for SIRT1 activation [34,35]**.** However, we did not observe the beneficial effects of treatment with a SIRT1 agonist (SRT1720) in improving the mitochondrial function in chagasic mice [36]. Thus, it is unlikely that benefits of PARP1 inhibition in rescuing mitochondrial oxidative metabolism in chagasic mice were delivered via increase in SIRT1 activity. Our finding of increased PARP1/PAR suggest that mitochondrial PARP1 may have had a direct effect on OXPHOS capacity through PARylation of various complexes of the electron transport chain in chagasic WT mice, as was observed in ovarian epithelial cancer cells [37].

The close proximity to the electron transport chain exposes the mtDNA to endogenous ROS, and thus, mtDNA may accumulate 20-100-fold higher level of oxidative adducts and DNA breaks than might be noted in nuclear DNA under similar conditions. Indeed, we have noted increased mtDNA damage in human cardiomyocytes *in vitro* infected with *T*. *cruzi* and in cardiac biopsies of chagasic patients [10]. Two mitochondrial enzymes, POLG and ExoG, carry out DNA base excision repair/single-strand breaks repair (BER/SSBR) of mtDNA [38]. POLG also assembles the mtDNA replisome, and carries the burden of mtDNA replication [38]. Whether PARP1 independently, or in conjunction with POLG and ExoG, is involved in mtDNA repair and replication in health or disease has not been studied so far. The first evidence that PARP1/PAR may exert a negative effect on mtDNA was provided by the observation of lower number of mtDNA mutagenic lesions in PARP1^-/-^ (vs. PARP1^+/+^) cells [39]. Szabo and coworkers showed that PARP1 depletion by shRNAi led to faster recovery of mtDNA integrity after initial oxidative insult in A549 epithelial cells [40]. In this study, we provide the first *in vivo* evidence that overexpression of PARP1 was detrimental to mtDNA maintenance and contributed to increased severity of chagasic cardiomyopathy. The PARylation of POLG or other proteins of the POLG replisome was not increased in chagasic heart, as was speculated to be the cause for deficiency of mtDNA repair in A549 cells exposed to oxidative stress [40]. PARP1 binding to LIG3 or other proteins of the DNA repair machinery or change in the expression of the components of the mtDNA replication and transcription machinery was also not noted in chagasic myocardium. Instead, we noted that PARP1 direct binding to POLG and mtDNA was increased and the levels of POLG and intact mtDNA were decreased in cardiac mitochondria of chagasic WT mice. Treatment of chagasic mice with PJ34 (PARP1 chemical inhibitor) or genetic depletion of PARP1 restored the POLG level and intact mtDNA content in chagasic heart. Further studies will be required to delineate if the PARP1 depletion restored the mtDNA repair or mtDNA replication activities of the POLG complex. Yet, our studies allow us to surmise that chemical inhibition of PARP1 by PJ34 treatment or genetic depletion of PARP1 was beneficial in controlling the mtROS and cardiac remodeling and restoring the mitochondrial oxidative metabolism as well as LV function in chagasic heart.

The pathologic features of the chronic Chagas cardiomyopathy also include focal areas of inflammation constituted by T cells and macrophages with a few eosinophils, plasma cells, and mast cells (reviewed in [6]). Parasites are rarely observed by conventional microscopic analysis of cardiac biopsies or random sections of explants or autopsy specimens during chronic Chagas disease [41]. It is, therefore, imperative that research community pays attention to host-derived factors as modulators of inflammatory responses in chronic Chagas disease. In this context, it is important to note that PARP1/PAR have been implicated in driving inflammation induced by cytotoxic agents (e.g. arsenite [42]). PARP1 suppression by genetic deletion or pharmacological inhibitors have been shown to be beneficial in reducing LPS-induced lung inflammation [43] and gut inflammation [44] in mice and humans. We have shown PARP1-dependent post-translational modification of Rel A (p65)-interacting nuclear proteins facilitated the assembly of NFκB transcription complex and cytokine gene expression in cardiomyocytes infected by *T*. *cruzi* [19]. Further, Sirtuin 1 (SIRT1), a highly conserved member of the family of NAD^+^-dependent Sir2 histone deacetylases, is known to compete with PARP1 for NAD^+^ substrate, and integrate mitochondrial metabolism and inflammation [45,46,47]. Treatment of mice with the SIRT1 agonist SRT1720 suppressed NFκB-dependent transcription and inflammatory stress in *T*. *cruzi*-infected cells and chagasic mice [36]. Thus, it is feasible that the observed benefits of PARP1 depletion in improving cardiac outcomes are not only due to improvement of mitochondrial function but also due to control of chronic inflammation in chagasic mice. We propose that while mitochondrial PARP1 is detrimental to maintaining the mtDNA integrity as observed in this study, nuclear PARP1 contributes to chronic inflammation in Chagas disease. Further studies will be required to delineate the potential role of nuclear PARP1 in driving chronic inflammation in Chagas disease.

In summary, we have used *in vitro* and *in vivo* models of *T*. *cruzi* infection and Chagas disease, and demonstrated that mitochondrial PARP1/PAR disturbs the POLG-dependent mtDNA integrity, and contributes to loss in mitochondrial function. Inhibition of mitochondrial PARP1/PAR offers a novel therapy in preserving the mitochondrial and LV function in chronic Chagas disease.

## Supporting information

S1 Table Oligonucleotides and antibodies used in this study. **(A)** Oligonucleotides for RT-qPCR. **(B)** Oligonucleotides for DNA amplification. **(C)** Antibodies.
(DOCX)

S2 Table Echocardiography in chronically infected WT, PARP1^+/-^, and PARP1^-/-^ mice. Mice were infected with *T*. *cruzi* (10,000 parasites per mouse). Vevo 2100 ultrasound system was used to perform transthoracic echocardiography in B and M mode and pulse-wave Doppler (PWD) echocardiography to assess the left ventricular and mitral valve functions at 150 days’ post-infection (n = 8–12 mice per group, three recordings per mouse). Data are presented as mean value ± SEM. Statistical significance is plotted as * (normal vs. infected) and ^#^ (PARP1^+/+^.*Tc* vs. PARP1^+/-^ .*Tc* or PARP1^-/-^.*Tc*) and presented as *^,#^ <0.05, **^,##^ p<0.01, ***^,###^ p<0.001.
(DOCX)

S1 Fig **(A) Genotyping of mice.** Total DNA was isolated from tail biopsies, and it was utilized as template in a PCR reaction with following oligonucleotides: Forward, 5’-CATGTTCGATGGGAAAGTCCC-3’; Reverse 1, 5’-CCAGCGCAGCTCAGAGAAGCCA-3’; and Reverse 2: 5’-AGGTGAGATGACAGGAGATC-3’. The F1/R1 oligonucleotides amplified a 112 bp band in WT (PARP1^+/+^) mice, and F1/R2 oligonucleotides amplified a 350 bp band in PARP1^-/-^ mice. Amplification of both bands indicated PARP1^+/-^ genotype. **(B) Confirmation of mitochondrial fractions purity by PCR**. Mitochondria Isolation Kit for Tissue (Abcam110168) was employed to isolate mitochondrial fractions. Total DNA from mitochondrial fractions was utilized as template in traditional PCR with gene-specific oligonucleotides to amplify for 28 cycles the 7S mtDNA fragment (184 bp) and *GAPDH* nuDNA fragment (101 bp). The PCR amplicons were resolved on 1.5% agrose gel. Note the amplification of nuDNA fragment was not noted in mitochondrial fractions.
(TIF)

S2 Fig **(A&B) The mtDNA level in PARP1**^**+/-**^ **chagasic mice.** Mice (WT and PARP1^+/-^) were infected with *T*. *cruzi* and monitored at 150 days’ post-infection. Representative gel images **(A**, n = 3 mice/group) show myocardial levels of 10 kb mtDNA and short 177-bp mtDNA and 96-bp nuDNA (*GAPDH*) fragments as controls. PCR amplification was performed for 28 cycles. Densitometry analysis was performed on PCR gels representing n≥ 6 mice/group, and density of the 10 kb mtDNA band, normalized against mtDNA and nuDNA fragments, is presented in **B.a&b. (C-H) Effect of PARP1 inhibitor on cardiomyocytes infected with *T*. *cruzi*.** Cardiac myocytes were infected with *T*. *cruzi* in presence or absence of PJ34 for 24 h. RT-qPCR was employed to evaluate the mRNA level for PARP1 and several components of the POLG replisome machinery, and data were normalized to GAPDH mRNA. **(I-K)** Cardiomyocytes were incubated for 24 with *Tc* in presence and absence of PJ34. ROS release was measured by an amplex red assay (**I**). MitoSOX red fluorescence detects mitochondrial O_2_^•−^ level (**J**). Ratio of fluorescence intensity of J-monomers (green) to J-aggregates (red) indicates mitochondrial depolarization (**K**). Data in C-K were acquired by using three biological replicates (duplicate analysis per sample). Data in all bar graphs are plotted as mean value ± SEM, and statistical significance are marked as *WT.*Tc* vs. WT, and ^#^WT.*Tc* vs. genetically-modified/infected or infected/PJ34-treated (^#^p<0.05, ***^,###^p<0.001).
(TIF)
