# Supplementary material for: PARP1 depletion improves mitochondrial and heart function in Chagas disease: Effects on POLG dependent mtDNA maintenance
Source: PLoS Pathog. 2018 May 31;14(5):e1007065. doi: 10.1371/journal.ppat.1007065 (PMC5979003; doi:10.1371/journal.ppat.1007065)
Supplement: S1 Table — (A) Oligonucleotides for RT-qPCR. (B) Oligonucleotides for DNA amplification. (C) Antibodies. (DOCX) [file ppat.1007065.s001.docx]

**S1 Table: Oligonucleotides and antibodies used in this study**

| **Gene Name** | **5‘-3’ Forward** | | **5‘-3’ Reverse** | | **Size (bp)** | **Accession#** | |
| --- | --- | --- | --- | --- | --- | --- | --- |
| **A: Oligonucleotides for RT-qPCR** | | | | | | | |
| *mATP6* | CCTTCCACAAGGAACTCCAA | | GGTAGCTGTTGGTGGGCTAA | | 187 | JF286601 | |
| *mATP8* | TGCCACAACTAGATACATCAACA | | GGGDTAATGAATGAGGCAAA | | 195 | NC_005089 | |
| *mCOI* | GCCTTTCAGGAATACCACGA | | AGGTTGGTTCCTCGAATGTG | | 234 | JF286601 | |
| *mCOIV* | ACTATGCTTTCCCCACTTACG | | CTTCTCCTTCTCCTTCAGCG | | 107 | NM_0099413 | |
| *mCOL1A1* | GAGCGGAGAGTACTGGATCG | | GCTTCTTTTCCTTGGGGTTC | | 158 | BC050014 | |
| *mCOL3A1* | GTCCACGAGGTGACAAAGGT | | GATGCCCACTTGTTCCATCT | | 204 | BC028248 | |
| *mCOL5A2* | GGTCCCTGACACACCTCAGT | | TGCTCCTCAGGAACCTCTGT | | 183 | BC055077 | |
| *mCYTB* | CTTACCCTTCATCCACACCTC | | GGGTGTTCTACAGGTTGTCC | | 129 | Z96064.1 | |
| *mND4* | GCATACCCCTTCATCCTTCTC | | GTGCTATGTGGCTAACTGAGG | | 115 | NC_0103391 | |
| *mPARP1* | GATCCCATCGACGTCAACTAC | | GATCACTTCCAGGTCATAGGC | | 141 | NM_0074152 | |
| *mPOLG* | AGGCACAGAACACATATGAGG | | TGCAAATCCCATTCTAGGTCC | | 139 | AB121698.1 | |
| *mPOLRMT* | TGCCTACATTTCCCACCTG | | CACCTCCCTGTACACATCTTG | | 145 | BC110697.1 | |
| *mSSBP1* | AATGAGATGTGGCGATCAGG | | TTCCACAAATATACGAGCCCC | | 150 | NM_2124683 | |
| *mTFB1M* | ATAGAGCCCAAGATCAAGCAG | | TGTAACAGCCTTCCAGTGC | | 146 | BC032930.1 | |
| *mTFB2M* | CATCTTGCATTCTAGGGTCCG | | CAAATCAGAATCAGGCAACGG | | 107 | BC138837.1 | |
| *mTOP1* | ACCATCTCCACAACGATTCC | | ATGCTTAGACTTTTCCCGGTC | | 143 | NM_0094082 | |
| *mGAPDH* | AACTTTGGCATTGTGGAAGG | | ACACATTGGGGGTAGGAACA | | 223 | NM_0080842 | |
| *hPARP1* | CCTGATCCCCCACGACTTTG | | CAGACCCTCCCCTGAGCAGA | | 134 | NM_001618 | |
| *hPOLG* | GTGGCTGTTTGAAGAGTTTGC | | GGTCAAGAGGTTGGTGATCTG | | 130 | BC050559.1 | |
| *hSSBP1* | AAGATCCCTGAATCGTGTGC | | CCTGATCGCCACATCTCATTAG | | 123 | BC093054.1 | |
| *hTOP1* | CTGTAGCCCTGTACTTCATCG | | TGGGTGTAGATTGATGTGCTC | | 122 | NM_003286.3 | |
| *hTFB1M* | AAGGAAATACTGCCATCGAGG | | AGAGTAGGGTCTATGTCTGCC | | 102 | BC017788.1 | |
| *hTFB2M* | CCAAGTAGAGGTGAGAAAAGGG | | AGGATCTGCCATTAGTTTCTGG | | 138 | BC003383.1 | |
| *hGAPDH* | CCCTTCATTGACCTCAACTACA | | ATGACAAGCTTCCCGTTCTC | | 101 | KJ891221.1 | |
| **B: Oligonucleotides for DNA amplification** | | | | | | | |
| *mLong mtDNA* | GCCAGCCTGACCCATAGCCATAATAT | | GAGAGATTTTATGGGTGTAATGCGG | | 10kb | JF286601.1 | |
| *m12SrDNA* | AGGTTTGGTCCTGGCCTTAT  GT | | GTGCTTGATACCCTCTCCTTAAA  GT | | 117 | V00665.1 | |
| *mGAPDH* | GGCAAAGTGGAGATTGTTG | | GTTGAATTTGCCGTGAGTG | | 96 | BC096042.1 | |
| *Tc18SrDNA* | TTTTGGGCAACAGCAGGTCT | | CTGCGCCTACGAGACATTCC | | 197 | X53917 | |
| Mouse (*m*), human (*h*) and *T. cruzi* (*Tc*) origin of genes is shown before gene name. | | | | | | | |
| **C: Antibodies** | | | | | | | |
| **Antigen** | | **Cat# (Clone#)** | | **Source** | | | **Host species** |
| AMP kinase α 1 (AMPK) | | sc19128 (C-20) | | Santa Cruz | | | Goat |
| Cytochrome oxidase I (COI) | | ab14705 (1D6E1A8) | | Abcam | | | Mouse |
| Cytochrome oxidase IV (COIV) | | ab16056 | | Abcam | | | Rabbit |
| DNA helicase Q4 (RECQL4) | | SAB1306892 | | Sigma | | | Rabbit |
| 5’-3’ DNA helicase (Twinkle or TWNK) | | ab187517 | | Abcam | | | Rabbit |
| DNA Ligase III (LIG3) | | ab185815 | | Abcam | | | Rabbit |
| DNA polymerase gamma (POLG) | | sc390634 (G-6) | | Santa Cruz | | | Mouse |
| DNA polymerase γ 2 POLG2 | | ab201100 | | Abcam | | | Rabbit |
| Glyceraldehyde 3 P dehydrogenase (GAPDH) | | 3683 (14C10, HRP-conjugate) | | Cell Signaling | | | Rabbit |
| Lamin B1 | | ab133741 (EPR8985B) | | Abcam | | | Rabbit |
| Poly(ADP-Ribose) (PAR) | | ALX-804-220 (10H) | | Enzo Life | | | Mouse |
| Poly(ADP-Ribose) Polymerase (PARP1) | | sc25780 (H-300) | | Santa Cruz | | | Rabbit |
| Ribonuclease H1 (RNASEH1) | | WH0246243M1 (5D10) | | Sigma | | | Mouse |
| Single-stranded DNA-binding protein 1 (SSBP1) | | sc67101 (FL-148) | | Santa Cruz | | | Rabbit |
| Transcription factor A, mitochondrial (TFAM) | | ab131607, HRP-conjugate | | Abcam | | | Rabbit |
| Transcription factor B2 mt (TFB2M) | | ab66014 | | Abcam | | | Rabbit |
| Transcription factor A, mitochondrial (TFA) | | ab138351 | | Abcam | | | Rabbit |
