# Supplementary material for: PARP1 depletion improves mitochondrial and heart function in Chagas disease: Effects on POLG dependent mtDNA maintenance
Source: PLoS Pathog. 2018 May 31;14(5):e1007065. doi: 10.1371/journal.ppat.1007065 (PMC5979003; doi:10.1371/journal.ppat.1007065)
Supplement: S2 Table — Mice were infected with T. cruzi (10,000 parasites per mouse). Vevo 2100 ultrasound system was used to perform transthoracic echocardiography in B and M mode and pulse-wave Doppler (PWD) echocardiography to assess the left ventricular and mitral valve functions at 150 days’ post-infection (n = 8–12 mice per group, three recordings per mouse). Data are presented as mean value ± SEM. Statistical significance is plotted as * (normal vs. infected) and # (PARP1+/+.Tc vs. PARP1+/- .Tc or PARP1-/-.Tc) and presented as *,# <0.05, **,## p<0.01, ***,### p<0.001. (DOCX) [file ppat.1007065.s002.docx]

**S2 Table. Echocardiography in chronically infected WT, PARP1^+/-^, and PARP1^-/-^ mice**

| **Parameters** | **Mode** | **WT** | **WT.*Tc*** | **PARP1^+/-^** | **PARP1^+/-^.*Tc*** | **PARP1^-/-^** | **PARP1^-/-^.*Tc*** |
| --- | --- | --- | --- | --- | --- | --- | --- |
| Left ventricular (LV) mass (mg) | M | 70.9±11.9 | 111.92±22.7** | 67.5±11.5 | 102.1±30 | 86.34±16.4 | 119.97±17.8 |
| internal diameter at systole (LVID-s, mm) | M | 1.4±0.2 | 3.2±0.2*** | 1.7±0.5 | 2.7±0.4^#^ | 2.10±0.29 | 2.3±0.3^##^ |
| LVID at diastole (LVID-d, mm) | M | 3.2±0.27 | 3.8±0.3 | 3.40±0.41 | 3.87±0.87 | 3.69±0.55 | 3.8±0.4 |
| % Fractional shortening (LVID-d – LVID-s x 100 / LVID-d) | M | 42.3±10.66 | 12.1±3.8*** | 33.02±3.05 | 18.60±3.13^#^ | 37.54±5.73 | 32.1±10.3^#^ |
| Area systole (mm^2^) | B | 9.17±3.9 | 17.97±3.03*** | 7.80±2.7 | 12.3±2.2^##^ | 11.67±4.28 | 11.98±1.4^##^ |
| Area diastole (mm^2^) | B | 14.3±3.9 | 24.3±7.7*** | 14.2±6.4 | 20.31±4.9^##^ | 16.14±4.28 | 11.98±1.4^##^ |
| Inter-ventricular septum (IVS-s, mm) | M | 0.9±0.2 | 1.1±0.35* | 0.75±0.05 | 0.99±0.07 | 0.81±0.3 | 1.0±0.1 |
| IVS-d (mm) | M | 0.6±0.1 | 0.97±0.3** | 0.70±0.12 | 0.85±0.04 | 0.77±0.14 | 0.9±0.3 |
| LV posterior wall (LVPW-s, mm) | M | 1.6±0.3 | 0.8±0.08*** | 1.26±0.2 | 1.3±0.15^##^ | 1.18±0.38 | 1.2±0.2^##^ |
| LVPW-d (mm) | M | 0.9±0.2 | 0.63±0.07** | 0.91±0.2 | 0.88±0.1^#^ | 0.76±0.23 | 0.9±0.1^##^ |
| IVS-s / LVPW-s ratio | M | 0.55±0.1 | 1.36±0.5*** | 0.61±0.14 | 0.77±0.1^###^ | 0.69±0.2 | 0.87±0.2^###^ |
| End systolic volume (ESV, µl) | B | 15.4±6.3 | 36.1±5.6*** | 17.6±2.6 | 24.8±4.0^##^ | 18.9±2.5 | 19.9±2.1^###^ |
| End diastolic volume (EDV, µl) | B | 50.2±13.5 | 52.3±58.2 | 58.2±4.4 | 43.2±2.9^#^ | 53.0±8.9 | 52.3±7.3 |
| Stroke volume (SV=EDV–ESV, µl) | B | 44.8±13.8 | 15.2±2.0*** | 38.5±5.4 | 21.28±4.8^##^ | 33.5±6.7 | 31.9±4.7^###^ |
| Cardiac output (HR x SV, ml/min) | B | 16.8±5.1 | 8.22±1.8*** | 19.95±4.27 | 11.72±2.3^##^ | 17.68±5.81 | 13.5±1.8^###^ |
| % Ejection fraction (EDV-ESV x 100 / EDV) | B | 65.5±7.6 | 35.1±5.5*** | 61.61±11.62 | 49.16±9.4^##^ | 61.79±8.89 | 56.5±9.0^###^ |
| Isovolumic contraction time (IVCT, ms) | PWD | 15.7±1.9 | 27.4±2.80*** | 16.7±1.7 | 15.5±3.5^###^ | 13.63±3.62 | 13.09±2.05^###^ |
| LV ejection time (LVET, ms) | PWD | 50.8±13.8 | 30.12±8.22*** | 47.6±10.0 | 36.97±7.8 | 45.37±12.38 | 47.14±12.8^##^ |
| IV relaxation time (IVRT, ms) | PWD | 21.7±7.5 | 32.5±11.10*** | 20.2±7.7 | 15.1±5.4^###^ | 23.50±7.29 | 21.05±8.6^##^ |
| LV Early (E) velocity (mm/s) | PWD | 751.2±122 | 447.9±85.3*** | 826.8±141.6 | 515.4±25.1 | 669.0±77.2 | 523.17±54.4^#^ |
| LV Late (A) velocity (mm/s) | PWD | 269.5±43.3 | 449.7±60.8*** | 319.8±103.5 | 201.1±28.3^###^ | 240.0±17.6 | 246.87±71^###^ |
| E/A ratio | PWD | 2.9±0.6 | 1.0±0.2*** | 2.8±1.05 | 2.61±0.4^###^ | 2.78±0.32 | 2.33±0.9^###^ |
| Myocardial performance (MPI=IVCT+IVRT/LVET) | PWD | 0.75±0.1 | 2.0±0.4*** | 0.83±0.28 | 0.90±0.3^###^ | 0.84±0.2 | 0.88±0.26^###^ |
| Mice were infected with *T. cruzi* (10,000 parasites per mouse). Vevo 2100 ultrasound system was used to perform transthoracic echocardiography in B and M mode and pulse-wave Doppler (PWD) echocardiography to assess the left ventricular and mitral valve functions at 150 days’ post-infection (n = 8-12 mice per group, three recordings per mouse). Data are presented as mean value ± SEM. Significance is plotted as * (normal vs. infected) and ^#^ (PARP1^+/+^.*Tc* vs. PARP1^+/-^ *.Tc* or PARP1^-/-^.*Tc*) and presented as *^,#^ <0.05, **^,##^ p<0.01, ***^,###^ p<0.001. | | | | | | | |
