# Supplementary figures and images for: PARP1 depletion improves mitochondrial and heart function in Chagas disease: Effects on POLG dependent mtDNA maintenance
Source: PLoS Pathog. 2018 May 31;14(5):e1007065. doi: 10.1371/journal.ppat.1007065 (PMC5979003; doi:10.1371/journal.ppat.1007065)

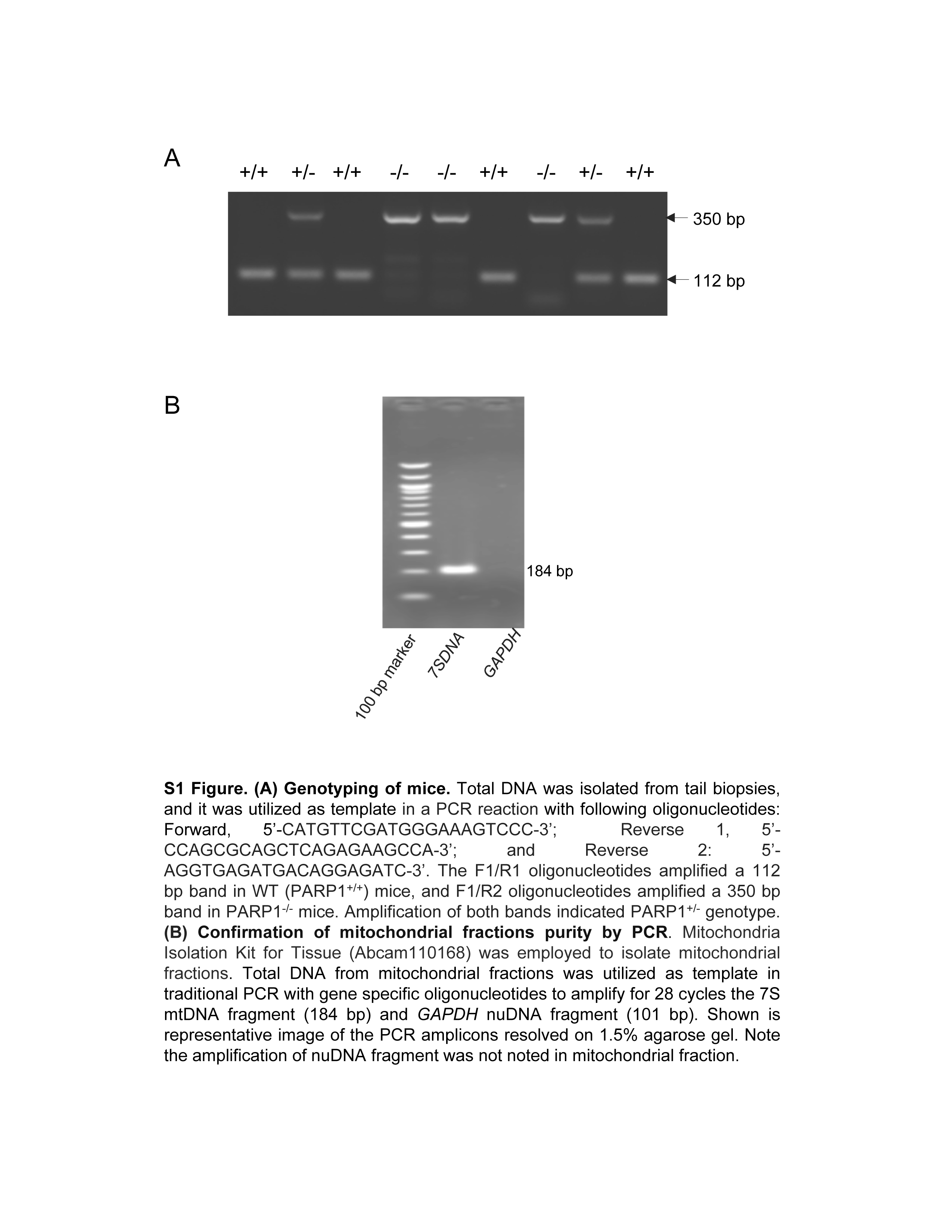

Supplement: S1 Fig — (A) Genotyping of mice. Total DNA was isolated from tail biopsies, and it was utilized as template in a PCR reaction with following oligonucleotides: Forward, 5’-CATGTTCGATGGGAAAGTCCC-3’; Reverse 1, 5’-CCAGCGCAGCTCAGAGAAGCCA-3’; and Reverse 2: 5’-AGGTGAGATGACAGGAGATC-3’. The F1/R1 oligonucleotides amplified a 112 bp band in WT (PARP1+/+) mice, and F1/R2 oligonucleotides amplified a 350 bp band in PARP1-/- mice. Amplification of both bands indicated PARP1+/- genotype. (B) Confirmation of mitochondrial fractions purity by PCR. Mitochondria Isolation Kit for Tissue (Abcam110168) was employed to isolate mitochondrial fractions. Total DNA from mitochondrial fractions was utilized as template in traditional PCR with gene-specific oligonucleotides to amplify for 28 cycles the 7S mtDNA fragment (184 bp) and GAPDH nuDNA fragment (101 bp). The PCR amplicons were resolved on 1.5% agrose gel. Note the amplification of nuDNA fragment was not noted in mitochondrial fractions. (TIF) [file ppat.1007065.s003.tif]

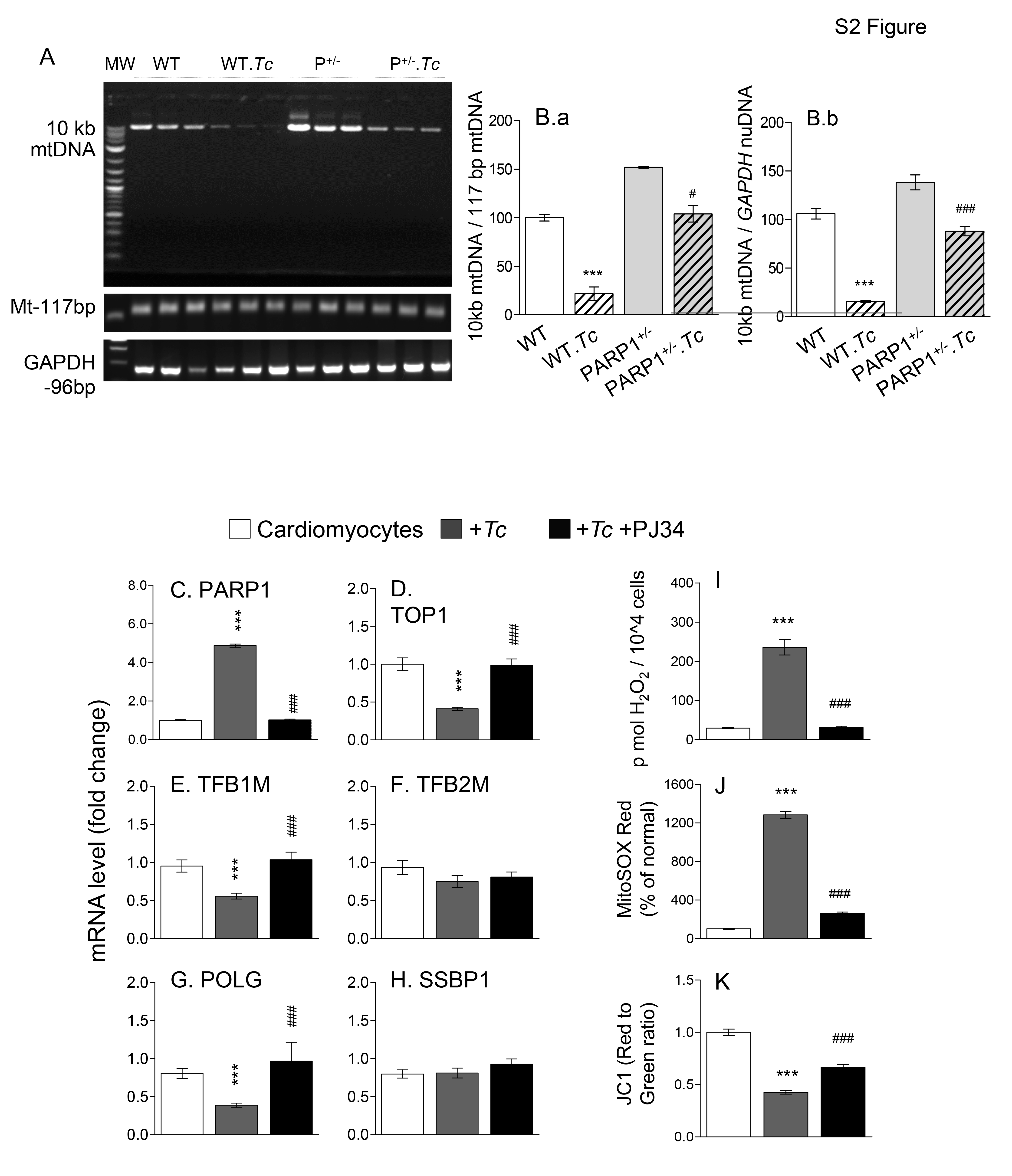

Supplement: S2 Fig — (A&B) The mtDNA level in PARP1+/- chagasic mice. Mice (WT and PARP1+/-) were infected with T. cruzi and monitored at 150 days’ post-infection. Representative gel images (A, n = 3 mice/group) show myocardial levels of 10 kb mtDNA and short 177-bp mtDNA and 96-bp nuDNA (GAPDH) fragments as controls. PCR amplification was performed for 28 cycles. Densitometry analysis was performed on PCR gels representing n≥ 6 mice/group, and density of the 10 kb mtDNA band, normalized against mtDNA and nuDNA fragments, is presented in B.a&b. (C-H) Effect of PARP1 inhibitor on cardiomyocytes infected with T. cruzi. Cardiac myocytes were infected with T. cruzi in presence or absence of PJ34 for 24 h. RT-qPCR was employed to evaluate the mRNA level for PARP1 and several components of the POLG replisome machinery, and data were normalized to GAPDH mRNA. (I-K) Cardiomyocytes were incubated for 24 with Tc in presence and absence of PJ34. ROS release was measured by an amplex red assay (I). MitoSOX red fluorescence detects mitochondrial O2•− level (J). Ratio of fluorescence intensity of J-monomers (green) to J-aggregates (red) indicates mitochondrial depolarization (K). Data in C-K were acquired by using three biological replicates (duplicate analysis per sample). Data in all bar graphs are plotted as mean value ± SEM, and statistical significance are marked as *WT.Tc vs. WT, and #WT.Tc vs. genetically-modified/infected or infected/PJ34-treated (#p<0.05, ***,###p<0.001). (TIF) [file ppat.1007065.s004.tif]
